# Macrophage-derived exosomal HMGB3 regulates silica-induced pulmonary inflammation by promoting M1 macrophage polarization and recruitment

**DOI:** 10.1186/s12989-024-00568-8

**Published:** 2024-03-07

**Authors:** Xiaofeng Qin, Zhiyuan Niu, Hui Chen, Yongbin Hu

**Affiliations:** 1 Department of Pathology, School of Basic Medical Science, Central South University, Changsha, China; 2 Department of Pathology, Xiangya Hospital, Central South University, Changsha, China

**Keywords:** HMGB3, Silicosis, Macrophage polarization, Inflammation

## Abstract

**Background:**

Chronic inflammation and fibrosis are characteristics of silicosis, and the inflammatory mediators involved in silicosis have not been fully elucidated. Recently, macrophage-derived exosomes have been reported to be inflammatory modulators, but their role in silicosis has not been explored. The purpose of the present study was to investigate the role of macrophage-derived exosomal high mobility group box 3 (HMGB3) in silica-induced pulmonary inflammation.

**Methods:**

The induction of the inflammatory response and the recruitment of monocytes/macrophages were evaluated by immunofluorescence, flow cytometry and transwell assays. The expression of inflammatory cytokines was examined by RT–PCR and ELISA, and the signalling pathways involved were examined by western blot analysis.

**Results:**

HMGB3 expression was increased in exosomes derived from silica-exposed macrophages. Exosomal HMGB3 significantly upregulated the expression of inflammatory cytokines, activated the STAT3/MAPK (ERK1/2 and p38)/NF-κB pathways in monocytes/macrophages, and promoted the migration of these cells by CCR2.

**Conclusions:**

Exosomal HMGB3 is a proinflammatory modulator of silica-induced inflammation that promotes the inflammatory response and recruitment of monocytes/macrophages by regulating the activation of the STAT3/MAPK/NF-κB/CCR2 pathways.

**Supplementary Information:**

The online version contains supplementary material available at 10.1186/s12989-024-00568-8.

## Introduction

Silicosis is an irreversible and fatal lung disease caused by long-term inhalation of silica (SiO_2_) dust and is characterized by chronic inflammation and fibrosis [1]. According to a document from the National Health Commission of China, there were 11,809 new cases of occupational pneumoconiosis in 2021 [2], and more than 450,000 pneumoconiosis patients are currently surviving. However, there are limited clinical treatments available for silicosis. Therefore, further exploration of the complex mechanism of silicosis is needed to develop a therapeutic strategy to mitigate its progression and reduce mortality.

Macrophages are the main effector cells of chronic inflammation. After silica dust exposure, the first critical step of host immune defence is the recognition and internalization of inhaled SiO_2_ by alveolar macrophages, which triggers pulmonary inflammation. Persistently activated macrophages cause inflammatory damage to lung tissue, ultimately leading to pulmonary fibrosis [3–5]. Previous studies have reported that intrapulmonary macrophages exhibit heterogeneity during the different stages of silicosis; the M1 subset is predominant in the early stage, and the M2 subset dominates the advanced fibrosis stage [6, 7].

Exosomes are membranous vesicles with a diameter of 30–150 nm that mediate local and distant cell-to-cell communication by carrying biological factors, including proteins, lipids, RNA and DNA [8, 9]. Increasing evidence has revealed the essential role of exosomes in the regulation of macrophage polarization, which contributes to the progression and outcome of many diseases, such as inflammation, tumours and metabolic diseases [8, 10–13]. Our previous study suggested that blocking exosome secretion alleviated lung inflammation and decreased the expression of IL-1β, IL-6 and TNF-α in bronchoalveolar lavage fluid in mice with silicosis [14]. Therefore, exosomes may be involved in the dysregulation of inflammation in silicosis, but the role of macrophage-derived exosomes in silica-induced inflammation remains largely unexplored.

High mobility group box 3 (HMGB3) is a nonhistone nucleoprotein belonging to the high mobility group box (HMGB) family that is highly expressed in embryos and has low expression in adult tissues. Aberrant upregulation of HMGB3 expression contributes to the progression of a variety of diseases, such as cancer and inflammation [15, 16]. HMGB3 is typically localized in the nucleus and binds to nucleosomes and nucleosome complexes in a sequence-independent manner, which affects DNA repair, replication, transcription and recombination [17, 18]. HMGB3 is involved in inflammatory cytokine induction as a universal sensor of nucleic acids during the activation of innate immune responses [19], and enhances the activation of innate immune response by regulating mitogen-activated protein kinase (MAPK) signalling pathways in Arabidopsis plants [20]. HMGB3 upregulation promotes inflammatory damage in intestinal epithelial cells in TNF-α-induced intestinal injury [16]. Moreover, HMGB3 can be packaged into nuclear exosomes (nEXOs) to regulate tumour angiogenesis [21]. Previous study has revealed that HMGB3 expression is upregulated in macrophage-derived exosomes after silica dust exposure [22]. Therefore, we hypothesized that exosomal HMGB3 might be involved in silica-induced lung inflammation by regulating inflammatory macrophage activation.

To investigate the role of macrophage-derived exosomal HMGB3 in silica-induced pulmonary inflammation, we constructed silica-exposed in vivo and in vitro models and found that the secretion of macrophage-derived exosomes was increased after silica exposure and strongly correlated with the inflammatory activation of monocytes/macrophages. Knockdown and overexpression functional rescue experiments showed that HMGB3 was a proinflammatory mediator found in macrophage-derived exosomes. In summary, we found that macrophages secreted HMGB3 within exosomes after silica exposure, which contributed to silica-induced inflammation by promoting M1 polarization and the recruitment of monocytes/macrophages. Therefore, the identification of exosomal HMGB3 as an important inflammatory mediator might provide a new strategy for attenuating inflammation in the early stage of silicosis.

## Materials and methods

### Cell lines and cell culture

A mouse leukemic macrophage line (RAW264.7) and a human monocytic cell line (THP-1) were obtained from the National Collection of Authenticated Cell Cultures (Shanghai, China). RAW264.7 cells were cultured in Dulbecco’s modified Eagle’s medium (DMEM; Gibco, Grand Island, NY, USA) supplemented with 10% foetal bovine serum (FBS; Gibco). A total of 7 × 10^5^ RAW264.7 macrophages or 1 × 10^7^ THP-1 monocytes were seeded in 10 cm cell culture dishes in 10 ml of conditioned medium supplemented with 10% FBS. Before silica dust exposure, THP-1 monocytes were cultured in RPMI 1640 medium (Gibco) supplemented with 10% FBS. THP-1 monocytes were first stimulated with 100 ng/ml phorbol-12-myristate-13-acetate (PMA; Sigma‒Aldrich, Merck KGaA, Darmstadt, Germany) for 24 h to induce differentiation into THP-1 macrophages, after which the cells were exposed to silica (SiO_2_, 12.5 µg/cm^2^; S5631, 1–5 μm; Sigma‒Aldrich), and after 36 h, the cell culture supernatant (SN) was harvested for exosome isolation. Similarly, the SN of RAW264.7 cells was collected for exosome isolation after 36 h of stimulation with SiO_2_ (25 µg/cm^2^). The undifferentiated THP-1 monocytes and untreated RAW264.7 macrophages were used as the M0 phenotype.

### Exosome isolation, identification and treatment

Differential centrifugation (Optimal^™^ L-80 XP, Beckman Coulter, CA, USA) was used to isolate exosomes from bronchoalveolar lavage fluid (BALF) or the SN of cultured cells. Exosome isolation was performed as previously described [14]. The total protein content of the exosomes was used to evaluate the quantity of exosomes and was measured by a micro-bicinchoninic acid (BCA) assay (Sigma‒Aldrich) or an ultraviolet spectrophotometer (Thermo Fisher Scientific, Massachusetts, USA).

To validate the exosomes, the expression of extracellular vesicle-related markers, including TSG101 (tumour susceptibility gene 101; 14497-1-AP, 1:1 000; Proteintech, Wuhan, China), HSP70 (heat shock protein 70; ab2787, 1:1 000; Abcam, Cambridge, UK) and CD63 (ab134045, 1:1000; Abcam), in the purified exosomes was examined by western blot analysis. Transmission electron microscopy (TEM; FEI, Massachusetts, USA) was used to observe the morphology of the exosomes, and nanoparticle tracking analysis (NTA; Zetasizer Nano ZS, Malvern Instruments, Worcestershire, UK) was used to determine the size distribution range of the exosomes.

For cell treatments, the purified exosomes were resuspended in sterile PBS, and the total protein content of the exosomes was measured by an ultraviolet spectrophotometer. Then, 50 µg of exosomes was used to treat 1 × 10^5^ RAW264.7 macrophage or 1 × 10^6^ THP-1 monocytes. In the SiO_2_ + GW4869-Exo treatment group, the same volume of exosomes as in the SiO_2_-Exo group was used.

To evaluate the activation of signalling pathways, a STAT3 inhibitor (Stattic, 5 µM; Abcam, USA) and an AKT inhibitor (MK2206, 10 nM; Beyotime, Shanghai, China) were used to inhibit protein phosphorylation.

### Exosome trafficking analysis in vitro and vivo study

To dynamically trace the exosomes, a PKH26 fluorescent kit (Sigma‒Aldrich) was used to label the exosomes according to the manufacturer’s instruction. In the in vivo experiment, PKH26-labelled exosomes were resuspended in 100 µl of sterile PBS and administered to C57BL/6 mice by tail vein injection. After 20 h, the distribution of exosomes was examined by an in vivo Xtreme II (BRUKER; Munich, Germany).

### Exosome secretion inhibition assay

A neutral sphingomyelinase inhibitor (GW4869, 10 µM; Cayman Chemical, Michigan, USA) was used to block exosome secretion. Before SiO_2_ exposure, THP-1 macrophages and RAW264.7 macrophages were pretreated with GW4869 (10 µM) for 24 h. The cells were then treated with a SiO_2_ suspension containing 10 µM GW4869 for 36 h, after which the cell culture supernatant was collected for exosome isolation. The inhibition of exosome secretion was evaluated by measuring the total protein concentration of the exosomes with a micro-BCA assay.

### Western blot analysis

Cells and exosome precipitates were lysed on ice for 30 min or 10 min, respectively, using RIPA lysis buffer supplemented with protease and phosphatase inhibitors. Then, the cell lysate was collected, ultrasonicated (exosome protein lysate was not ultrasonicated), and centrifuged at 4 °C and 12,000 rpm for 12 min, after which the supernatant was harvested. The total protein concentration was assessed by a micro-BCA assay. 30 µg of total protein was subjected to SDS‒PAGE and detected with antibodies. The primary antibodies used for western blot analysis were as follows: anti-CD68 (28058-1-AP, 1:1000; Proteintech, Wuhan, China), anti-CD31 (28083-1-AP, 1:1000; Proteintech), anti-SP-B (sc-133,143, 1:1000; Santa Cruz Biotechnology, Texas, USA), anti-podoplanin (PDPN; sc-53,533, 1:1000; Santa Cruz Biotechnology), anti-caveolin-1 (sc-53,564, 1:1000; Santa Cruz Biotechnology), anti-p-STAT1 (Tyr701) (340,797, 1:1000; Zenbio, Chengdu, China), anti-p-STAT3 (AP0247, 1:1000; Bioworld, Nanjing, China), anti-p-AKT (ab81283, 1:500; Abcam, Cambridge, UK), anti-p-NF-κB p65 (310,013, 1:1000; Zenbio), anti-p-ERK1/2 (AF1015, 1:1000; Affinity Biosciences, Jiangsu, China), anti-p-p38 MAPK (ab4822, 1:1000; Abcam), anti-STAT1 (ab4822, 1:1000; Proteintech), anti-STAT3 (10253-2-AP, 1:1000; Proteintech), anti-AKT (10176-2-AP, 1:1000; Proteintech), anti-NF-κB p65 (R25149, 1:1000; Zenbio), anti-ERK1/2 (BF8004, 1:1000; Affinity Biosciences), anti-p38 MAPK (R25239, 1:1000; Zenbio), anti-IL-1β (ab283818, 1:1000; Abcam), anti-HMGB1 (R22773, 1:1000; Zenbio), anti-HMGB2 (R26860, 1:1000; Zenbio), anti-HMGB3 (D160490, 1:1000; Sangon Biotech, Shanghai, China; ab75782, 1:1000; Abcam), anti-beta-actin (β-actin; 20536-1-AP, 1:1000; Proteintech), anti-Histone-H3 (17168-1-AP, 1:1000; Proteintech) and anti-glyceraldehyde 3-phosphate dehydrogenase (GAPDH; 60004-1-AP, 1:1000; Proteintech). After incubation of the corresponding HRP-conjugated secondary antibody, a chemiluminescent system (ChemiDocTM XRS+, Bio-Rad, USA) was used for detection.

### Reverse transcription‒polymerase chain reaction (RT–PCR)

Total RNA was extracted using TRIzol, 1 µg of total RNA was reverse transcribed into cDNA, and qPCR was performed according to the manufacturer’s instructions (SureScript™ First-Strand cDNA Synthesis Kit; BlazeTaq™ SYBR Green qPCR Mix 2.0; Genecopoeia, USA). β-Actin or GAPDH was used as a reference gene. The primer sequences are listed in Table S1.

### Animal model

A 28-day silicosis mouse model was constructed by a single intratracheal injection of a silica suspension (100 mg/kg body weight), and the BALF and lungs were harvested for further analysis.

Fifteen mice were randomly divided into three groups with 5 mice in each group. The exosomes (7.5 µg/g body weight) were first suspended in 50 µl of PBS and administered to each animal by intratracheal injection. Exosomes (10 µg/g of body weight per two days) were subsequently administered to the mice by tail vein injection until they were sacrificed on Day 9, at which point the lungs were collected for flow cytometric analysis. An equal volume of PBS was administered to mice in the control group. The exosomes used were derived from RAW264.7 macrophages. We transfected RAW264.7 macrophages with siRNA and isolated exosomes from the cell culture supernatant, resulting in exosomes derived from siNC-transfected SiO_2_-exposed RAW264.7 macrophages (SiO_2_ + siNC-Exo) and exosomes derived from siHMGB3-transfected SiO_2_-exposed RAW264.7 macrophages (SiO_2_ + siHMGB3-Exo). The animal protocols were in accordance with the requirements of related regulations and procedures of the National Institutes of Health Guide for the Care and Use of Laboratory Animals, as well as ethical principles.

### Immunohistochemical staining

The paraffin sections were dewaxed, hydrated, and repaired with EDTA antigen retrieval solution under high-pressure steam for 10 min. Then, the sections were blocked with catalase for 15 min, washed 3 times with PBS, and sealed with normal goat serum for 30 min. Next, the sections were incubated with primary antibodies at 4 °C overnight. The sections were washed 3 times with PBS, incubated with anti-mouse/anti-rabbit IgG for 30 min, washed 3 times with PBS, stained with DAB for 5 min, washed with running water for 3 min, and stained with haematoxylin for 30 s. After the sections were washed with running water for 3 min, the staining was observed under a microscope, followed by gradient dehydration and preservation with neutral balsam. Anti-CD68 (1:200, 28058-1-AP; Proteintech), anti-HMGB3 (1:100, D160490; Sangon Biotech; 1:100, ab75782; Abcam) and anti-α-SMA (1:200, 14395-1-AP; Proteintech) antibodies were used for immunohistochemical staining.

### Immunofluorescence analysis

Before performing immunofluorescence analysis, the suspended THP-1 monocytes were collected to prepare cell smears. Then, the cell smears and adherent cells grown on glass coverslips were fixed with 4% paraformaldehyde for 30 min, permeabilized with 0.2% Triton X-100 for 5 min, blocked with 3% BSA-PBS for 30 min, and subsequently labelled with anti-iNOS (1:100, 53-5920-82; Invitrogen) antibodies at 4 °C overnight. DAPI was used to stain the nuclei. Fluorescence was observed with an inverted fluorescence microscope (Olympus, Tokyo, Japan).

### Flow cytometry

For flow cytometry, the cells were collected, washed 3 times with PBS and subsequently fixed with ice-cold methyl alcohol on ice for 30 min. After being washed 3 times with PBS, the cells were stained with anti-iNOS (0.125 µg, 2,366,416; Invitrogen) antibodies at 4 °C for 2 h. After being washed 3 times with PBS, the cells were resuspended in 500 µl of PBS, and examined by a BD FACSAria™ Fusion (Becton, Dickinson and Company, USA).

For lung tissue analysis, cardiopulmonary lavage was performed with PBS containing 0.5 M EDTA to remove residual blood from the lung tissue, and the tissue was then digested with 2 mg/ml collagenase IV at 37 °C for 30 min with mixing (125 rpm/min). After serum was added to terminate the digestion, the cells were filtered through a nylon strainer with a pore size of 70 μm and then centrifuged at 500 × g for 5 min. Red blood cell lysis buffer was used to lyse the red blood cells, after which the cells were washed 3 times with PBS. The dead and live cells were labelled with the fixable viability dye efluor™ 506 (0.2 µg, 2,443,412; Invitrogen), fixed on ice with fixation solution for 25 min, stained with CD45 (0.2 µg, 557,659; BD Pharmingen™), CD11b (0.2 µg, 2,416,225; Invitrogen) and F4/80 (0.2 µg, 2,430,442; Invitrogen) antibodies at room temperature for 30 min, permeabilized for 5 min, and then stained with iNOS (0.25 µg, 2,366,416; Invitrogen) and CD206 (0.2 µg, 2,506,988; Invitrogen) antibodies at room temperature for 30 min. After being washed 3 times with PBS, the cells were resuspended in 500 µl of PBS and examined by a BD FACSAria™ Fusion.

### Enzyme-linked immunosorbent assay (ELISA)

ELISA kits were used to analyse the expression levels of murine IL-1β, IL-6 and TNF-α in the cell culture supernatant or bronchoalveolar lavage fluid according to the instructions. The ELISA kits were obtained from Proteintech.

### Cell migration assay

To perform the transwell assay, THP-1 monocytes and RAW264.7 macrophages were collected and resuspended in FBS-free conditioned medium. Then, 150 µl of the cell suspension (1–2 × 10^5^ cells) was added to the upper chamber of the transwell chamber (3422; Corning CoStar, New York, USA) with a pore size of 8 μm, and 700 µl of conditioned medium supplemented with 10% FBS was added to the lower chamber.

Purified exosomes were resuspended in sterile PBS, and the total protein content of the exosomes was measured by an ultraviolet spectrophotometer. Then, 150 µg of exosomes (NC-Exo and SiO_2_-Exo) was mixed with conditioned medium supplemented with 10% FBS to a total volume of 700 µl, and the mixture was added to the lower chamber and incubated. In the SiO_2_ + GW4869-Exo treatment group, an equal volume of exosomes as in the SiO_2_-Exo group was used for treatment. The control group was treated with the same volume of sterile PBS.

After 24 h of incubation, the transwell chambers were harvested, fixed with 4% paraformaldehyde at room temperature for 25 min and stained with crystal violet (C0121; Beyotime, Shanghai, China) for 15 min; then, ImageJ software was used to analyse the results.

THP-1 monocytes and RAW264.7 macrophages were pretreated with different concentrations of the C-C motif chemokine receptor 2 (CCR2) antagonist (N-(2-(3-((4-hydroxy-4-(5-(pyrimidin-2-yl)pyridin-2-yl)cyclohexyl)amino)pyrrolidin-1-yl)-2-oxoethyl)-3-(trifluoromethyl)benzamide; C_29_H_31_F_3_N_6_O_3_; PF-4,136,309; Catalog No. A3495; APExBIO, Texas, USA) (10 nM, 20 nM, 40 nM, or 100 nM) for 20 min. Then, 150 µl of the cell suspension (1–2 × 10^5^ cells) was added to the upper chamber of the transwell chamber with the CCR2 antagonist (a final concentration of 10 nM, 20 nM, 40 nM, or 100 nM), and 700 µl of conditioned medium supplemented with 10% FBS (mixed with 150 µg of SiO_2_-Exo from each group) was added to the lower chamber. After 24 h of incubation, the transwell chambers were harvested and analysed as previously described.

### Plasmid construction and transfection

Murine HMGB3 cDNA was cloned and inserted into the pcDNA3.1(+) vector at the Pme I and Not I sites. A total of 1.5 µg of plasmid was mixed with 3 µl of Lipofectamine 2000 (Invitrogen, Thermo Fisher Scientific, Massachusetts, USA) in 100 µl of OPTI-MEM (Gibco), and the mixture was used to transfect cells (1.5 µg of plasmid per 10^5^ cells) for 24–36 h.

### RNA interference

Three siRNAs against murine HMGB3 and their corresponding negative controls were constructed and generated by RiboBio (Guangzhou, China). The siRNA sequences were as follows: si-HMGB3#1, CATGCAGGGAAGAACATAA; si-HMGB3#2, GGCAGATAAAGTCCGATAT; and si-HMGB3#3, AGCAGCCTTATGTCACCAA. The siRNAs (100 nM) were mixed with the transfection reagent (RiboBio; Guangzhou, China), and the mixture was used to transfect cells for 24–36 h according to the manufacturer’s instructions. Short hairpin RNAs (shRNAs) targeting human HMGB3 and their negative controls were generated by RiboBio (Guangzhou, China). The following shRNA sequences were used: shHMGB3#1: gatcccAAGGAAAGTTTGATGGTGCAActcgagTTGCACCATCAAACTTTCCTTtttttggat; shHMGB3#2: gatcccGGCTCCATCATGATCTTCGACGATActcgagCCGAGGTAGTACTAGAAGCTGCTATtttttggat; and shHMGB3#3: gatcccGCAGATAAAGTGCGCTATGATctcgagCGTCTATTTCACGCGATACTAtttttggat.

### Statistical analysis

GraphPad Prism software (La Jolla, CA, USA) was used to analyse the data. The results are expressed as the mean ± SEM. For numerical data, Student’s *t* test (unpaired, two-tailed) was used for comparisons between two groups, and two-way ANOVA followed by Tukey’s multiple comparisons test was used for multiple comparisons. A value of *P* < 0.05 indicated statistical significance.

## Results

### The quantity of exosomes secreted by macrophages was significantly increased in mice with silicosis

**Fig. 1:**
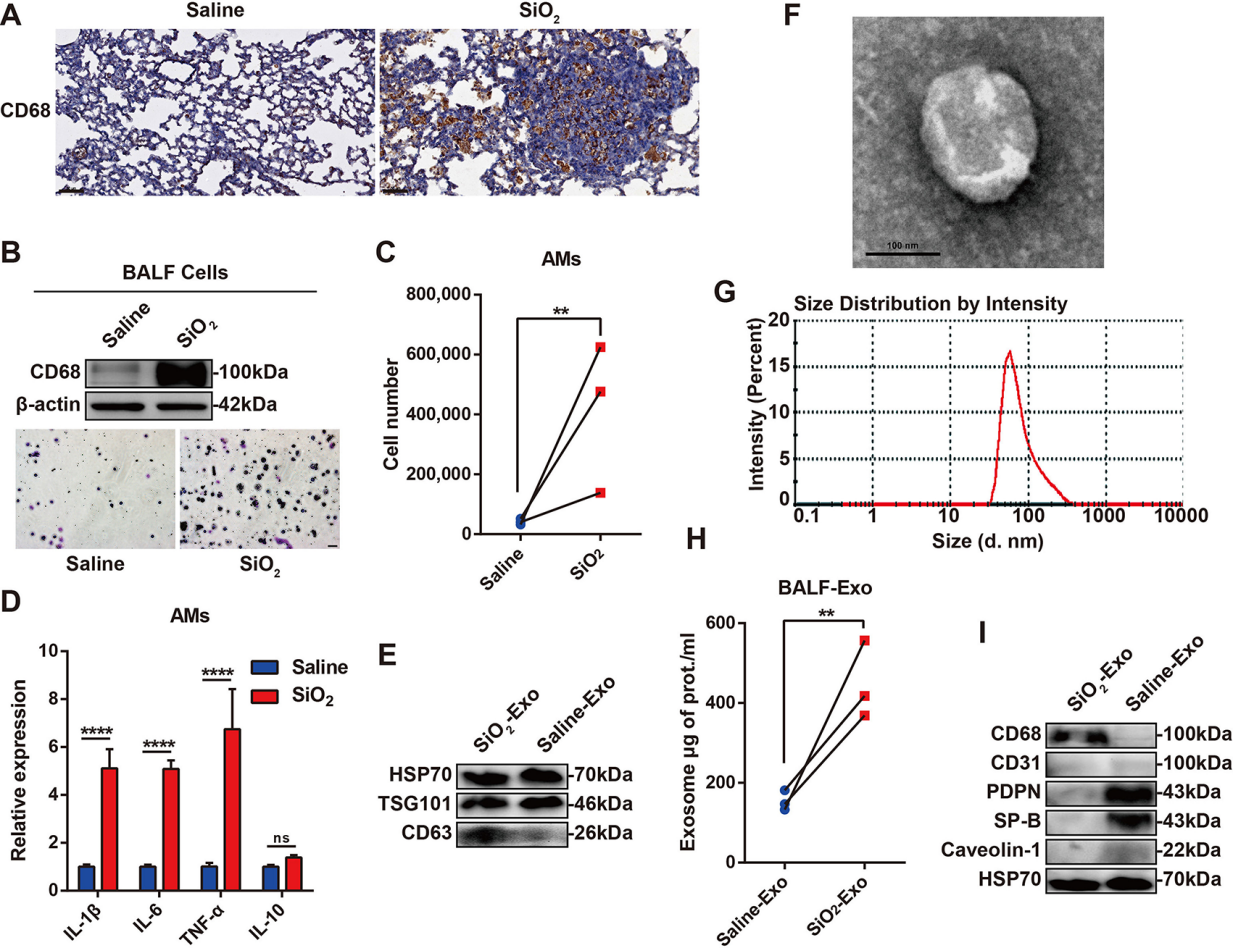
The number of exosomes secreted by alveolar macrophages is significantly increased in the BALF of mice with silicosis. **(A)**. Representative image of CD68 expression in the lung tissue of mice treated with saline or silica dust and examined by immunohistochemical staining (scale bar = 50 μm). **(B-D)**. After 28 days of exposure to silica dust, BALF was collected, the morphology of the cells in BALF was observed by Giemsa staining (scale bar = 20 μm), and CD68 expression in these cells was examined by western blot analysis **(B)**. The number of AMs in the BALF **(C)** and the expression of IL-1β, IL-6 and TNF-α in AMs were examined by RT–PCR **(D)**. *n* = 5 mice per group. Saline = saline-treated mice; silica = silica dust-treated mice. **(E-H)**. Exosomes were isolated from the BALF of mice treated with saline or silica dust, the expression of exosome-related markers (HSP70, TSG101 and CD63) was examined by western blot analysis **(E)**, exosome morphology was observed by TEM (scale bar = 100 nm) **(F)**, the size distribution of the exosomes was analysed by NTA **(G)**, and the total exosomal protein concentration was examined by a micro-BCA assay **(H)**. *n* = 15 mice per group. **(I)**. Representative image showing the expression of CD68, CD31, PDPN, SP-B, caveolin-1 and HSP70 in exosomes derived from BALF and measured by western blot analysis. *n* = 15 mice per group. The data are representative of three individual experiments and expressed as the mean ± SEM. The data were analysed by two-tailed Student’s *t* test or two-way ANOVA. *P < 0.05, **P < 0.01, ***P < 0.001, ****P < 0.0001, ns = not significant. *Abbreviations* SiO_2_ = silica dust; BALF = bronchoalveolar lavage fluid; AMs = alveolar macrophages; TEM = transmission electron microscope; NTA = nanoparticle tracking analysis

To verify the distribution of macrophages, we constructed a 28-day silicosis mouse model and examined CD68 (a macrophage marker) expression in the lung tissue. Compared with that in control mice, CD68 expression was markedly upregulated in the lung tissue of mice with silicosis (Fig. 1A). Moreover, the cells in BALF were collected for counting and Giemsa staining, and the results showed that the number and proportion of alveolar macrophages (AMs) were significantly increased in the BALF of mice with silicosis (Fig. 1B-C). RT‒PCR showed that inflammatory cytokines (IL-1β, IL-6 and TNF-α) were upregulated in AMs, but IL-10 expression was not significantly different (Fig. 1D), which indicated that AMs in the early stage of silicosis were mainly inflammatory macrophages.

We next explored the role of macrophage-derived exosomes in silicosis-related inflammation. We collected BALF and extracted exosomes from the fluid by differential centrifugation. Western blot analysis showed that exosome-related markers (HSP70, TSG101 and CD63) were highly expressed in the extracted exosomes (Fig. 1E). We used transmission electron microscopy (TEM) to observe the morphology of the exosomes and found that the exosomes were membrane-like structures with a “cup shape” (Fig. 1F). Nanoparticle tracking analysis (NTA) showed that the purified exosomes were between 30 and 150 nm in size, with a peak value of 83.9 nm (Fig. 1G). The total protein concentration of the exosomes was examined by a micro-BCA assay, which was used to evaluate the quantity of the exosomes. Compared with that in control mice, exosome secretion in the BALF of mice with silicosis was significantly increased (Fig. 1H). Exosomes typically carry marker molecules from their source cells. To identify the main source cells of the exosomes in BALF, we examined the expression of a macrophage-related marker (CD68), a vascular endothelial cell-related marker (CD31) and alveolar epithelial cell-related markers (PDPN, SP-B and caveolin-1) in the exosomes. The results indicated that the secreted exosomes in the BALF of mice with silicosis were mainly derived from macrophages (Fig. 1I). Exosomes in the saline-treated group (control) were mainly derived from alveolar epithelial cells (Fig. 1I). These results showed that exosomes derived from macrophages were significantly increased in mice with silicosis.

### The quantity of exosomes secreted by SiO_2_-exposed macrophages increased significantly in vitro

**Fig. 2:**
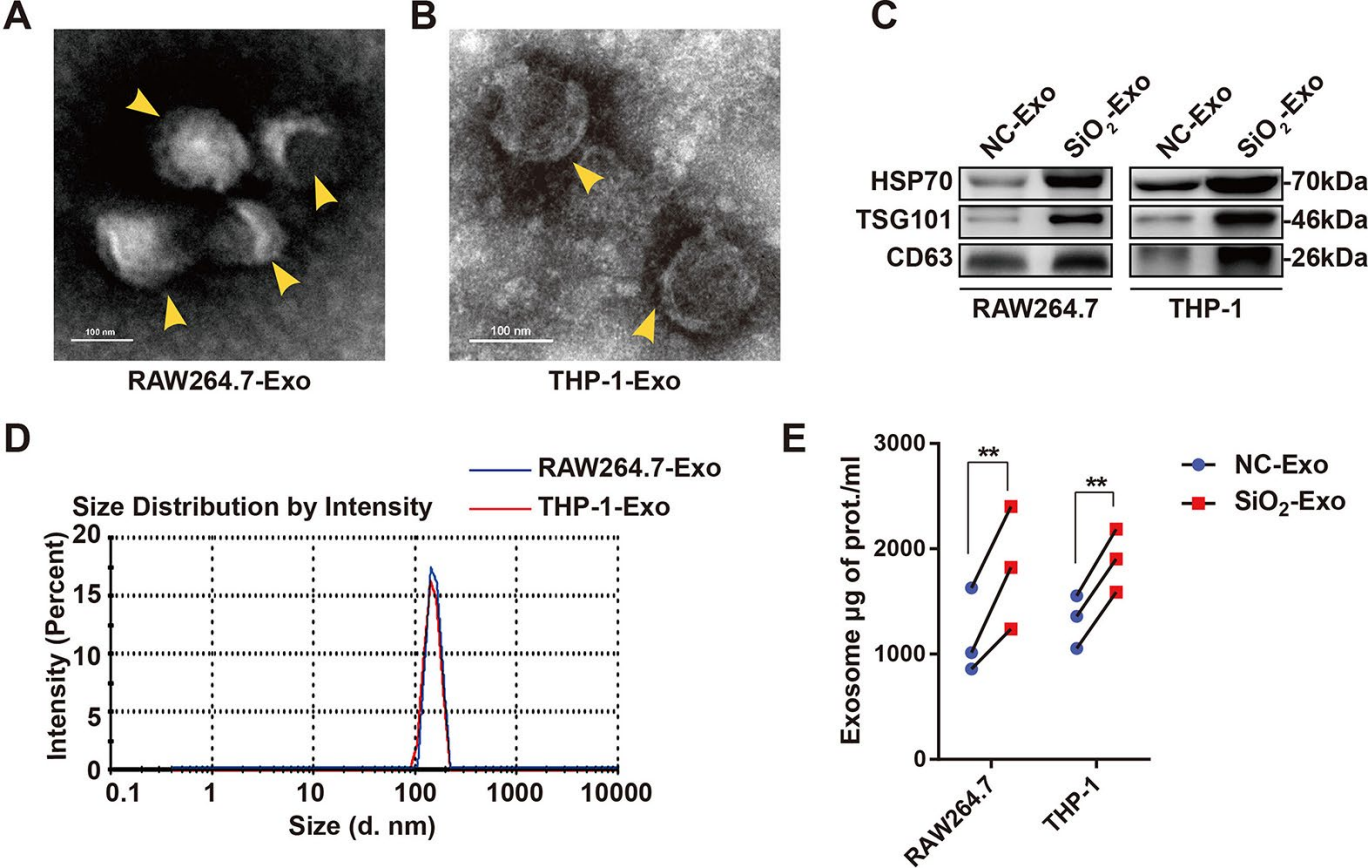
The secretion of exosomes by macrophages is increased by silica exposure. **(A-B)**. The morphology of exosomes derived from the supernatant of RAW264.7 macrophages and THP-1 macrophages treated with silica dust was observed by TEM. The yellow arrowheads indicate exosomes. The scale bar represents 100 nm. **(C)**. Representative western blot image showing the expression of HSP70, TSG101 and CD63 in exosomes derived from RAW264.7 macrophages and THP-1 macrophages with or without SiO_2_ exposure; equal volumes of exosomes (in a total volume of 30 µl) were subjected to SDS–PAGE. **(D)**. NTA showing the size distribution of exosomes derived from RAW264.7 macrophages and THP-1 macrophages treated with SiO_2_. **(E)**. Micro-BCA assay analysis of the total protein content of exosomes derived from RAW264.7 macrophages and THP-1 macrophages treated with or without SiO_2_ for 36 h. *n* = 3 per group. The data are representative of three individual experiments and were analysed by two-way ANOVA. *P < 0.05, **P < 0.01. *Abbreviations* SiO_2_ = silica dust; NC-Exo = exosomes derived from cells without SiO_2_ exposure; SiO_2_-Exo = exosomes derived from SiO_2_-exposed macrophages; TEM = transmission electron microscopy; NTA = nanoparticle tracking analysis; BCA = bicinchoninic acid

Next, we investigated the secretion of exosomes by SiO_2_-exposed macrophages in vitro. Macrophages were exposed to SiO_2_ for 36 h, after which the cell culture supernatant (SN) was harvested. Exosomes in the SN were subsequently extracted by differential centrifugation. TEM showed that the exosomes exhibited a membrane-like structure with a diameter of 60–100 nm (Fig. 2A-B, yellow arrowheads). Western blot analysis revealed that the extracted exosomes highly expressed HSP70, TSG101 and CD63 (Fig. 2C). NTA showed that the particle sizes of the purified exosomes were mainly distributed in the range of 80–200 nm, with peaks at 152.5 nm and 149.4 nm (Fig. 2D). The total protein concentration of the exosomes was determined by a micro-BCA assay, and the quantity of exosomes secreted by SiO_2_-exposed macrophages was significantly increased (Fig. 2E).

### SiO_2_-Exo promoted the polarization of M0 macrophages to M1 macrophages in vitro

**Fig. 3:**
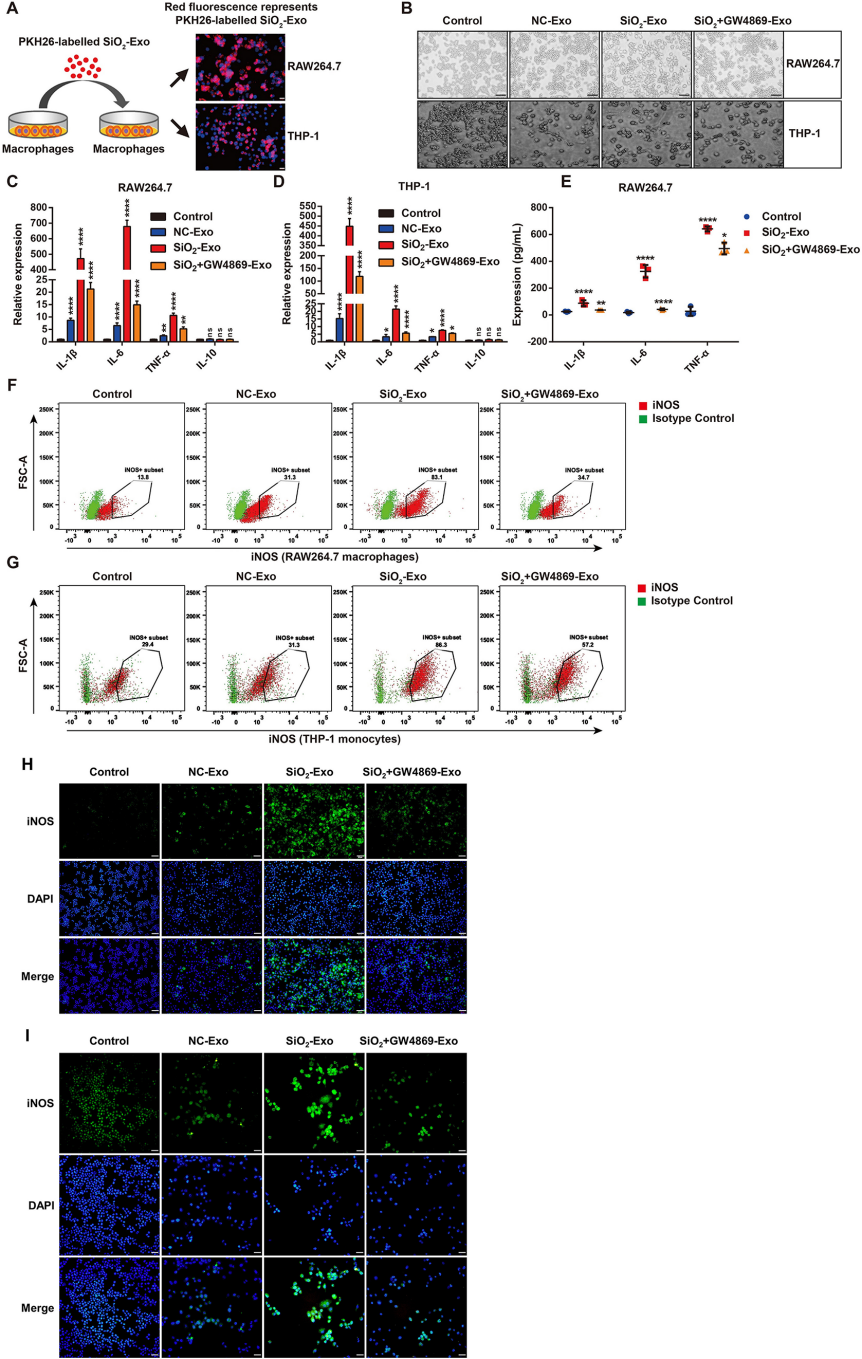
Exosomes derived from SiO_2_-exposed macrophages promote inflammatory activation in monocytes/macrophages **(A)** The purified exosomes were labelled with PKH26 dye and then added to RAW264.7 macrophages and THP-1 macrophages. The uptake of PKH26-labelled exosomes was observed by fluorescence microscopy. The scale bar represents 20 μm. **(B)** Representative image of the morphological changes in RAW264.7 macrophages and THP-1 monocytes treated with PBS, NC-Exo, SiO_2_-Exo or SiO_2_ + GW4869-Exo. The scale bar represents 50 μm. **(C-D)**. RT–PCR analysis of the expression of IL-1β, IL-6, TNF-α and IL-10 in RAW264.7 macrophages and THP-1 monocytes treated with PBS, NC-Exo, SiO_2_-Exo or SiO_2_ + GW4869-Exo. **(E)**. ELISA analysis of the levels of proinflammatory factors in the supernatant of RAW264.7 macrophages treated with PBS, SiO_2_-Exo or SiO_2_ + GW4869-Exo. *n* = 3 each group. **(F-G)**. Flow cytometric analysis of the proportions of iNOS^+^ cells among RAW264.7 macrophages and THP-1 monocytes after PBS, NC-Exo, SiO_2_-Exo or SiO_2_ + GW4869-Exo treatment. **(H-I)**. Immunofluorescence analysis of iNOS expression in RAW264.7 macrophages **(H)** and THP-1 monocytes **(I)** after PBS, NC-Exo, SiO_2_-Exo or SiO_2_ + GW4869-Exo treatment. The scale bar represents 50 μm. The data are representative of three individual experiments and expressed as the mean ± SEM. The data were analysed by two-way ANOVA. *P < 0.05, **P < 0.01, ***P < 0.001, ****P < 0.0001, ns = not significant. *Abbreviations* SiO_2_ = silica dust; NC-Exo = exosomes derived from cells without SiO_2_ exposure; SiO_2_-Exo = exosomes derived from SiO_2_-exposed macrophages; SiO_2_ + GW4869-Exo = exosomes derived from SiO_2_-exposed macrophages treated with GW4869 (10 µM)

We next investigated the role of SiO_2_-exposed macrophage-derived exosomes (SiO_2_-Exo) in silica-induced inflammation. First, we aimed to verify whether exosomes could be taken up by macrophages. PKH26 dye was used to label exosomes derived from SiO_2_-exposed macrophages, and these exosomes were cocultured with M0 macrophages for 12 h. The results showed that red fluorescence was visible in the membrane and cytoplasm of macrophages, which indicated that the exosomes were taken up by the macrophages (Fig. 3A).

To explore whether SiO_2_-Exo regulated the inflammatory activation of macrophages, we isolated exosomes from macrophages with or without SiO_2_ exposure, and GW4869 (10 µM) was used to block exosome secretion by SiO_2_-exposed macrophages. The results showed that GW4869 could effectively inhibit exosome secretion by SiO_2_-exposed macrophages (Figure S1). These exosomes were cocultured with M0 RAW264.7 macrophages or THP-1 monocytes for 36 h, after which changes in cell morphology were observed under a microscope. In the SiO_2_-Exo treatment group, the morphology of THP-1 monocytes changed from a suspension state to an adherent state, and a large number of THP-1 cells and RAW264.7 cells exhibited polygonal morphology (Fig. 3B), which was consistent with M1 macrophages. The number and proportion of cells with polygonal morphology were decreased in the NC-Exo treatment group and SiO_2_ + GW4869-Exo treatment group (Fig. 3B). RT‒PCR indicated that SiO_2_-Exo markedly upregulated the expression of IL-1β, IL-6 and TNF-α, but IL-10 expression did not significantly differ. After using GW4869 to block exosome secretion by SiO_2_-exposed macrophages (SiO_2_ + GW4869-Exo treatment group), the expression of IL-1β, IL-6 and TNF-α was significantly downregulated compared with that in the SiO_2_-Exo treatment group (Fig. 3C-D). ELISA confirmed these results (Fig. 3E).

Flow cytometry revealed that the proportions of iNOS^+^ cells were markedly increased in the SiO_2_-Exo treatment group (83.1% in RAW264.7 cells and 86.3% in THP-1 cells) but were decreased in the SiO_2_ + GW4869-Exo treatment group (34.7% in RAW264.7 cells and 57.2% in THP-1 cells) (Fig. 3F-G). Immunofluorescence analysis of THP-1 cells revealed that SiO_2_-Exo induced CD68 expression, promoted the differentiation of monocytes into macrophages (Figure S2A). SiO_2_-Exo upregulated iNOS expression in RAW264.7 macrophages and THP-1 macrophages, while iNOS expression was decreased in the SiO_2_ + GW4869-Exo treatment group (Fig. 3H-I). Moreover, the CCK-8 assay showed that SiO_2_-Exo had no effect on the proliferation of RAW264.7 macrophages (Figure S2B). These results suggested that SiO_2_-Exo played a role in the inflammatory responses induced by silica.

### SiO_2_-Exo promoted the migration of monocytes/macrophages via CCR2 in vitro

**Fig. 4:**
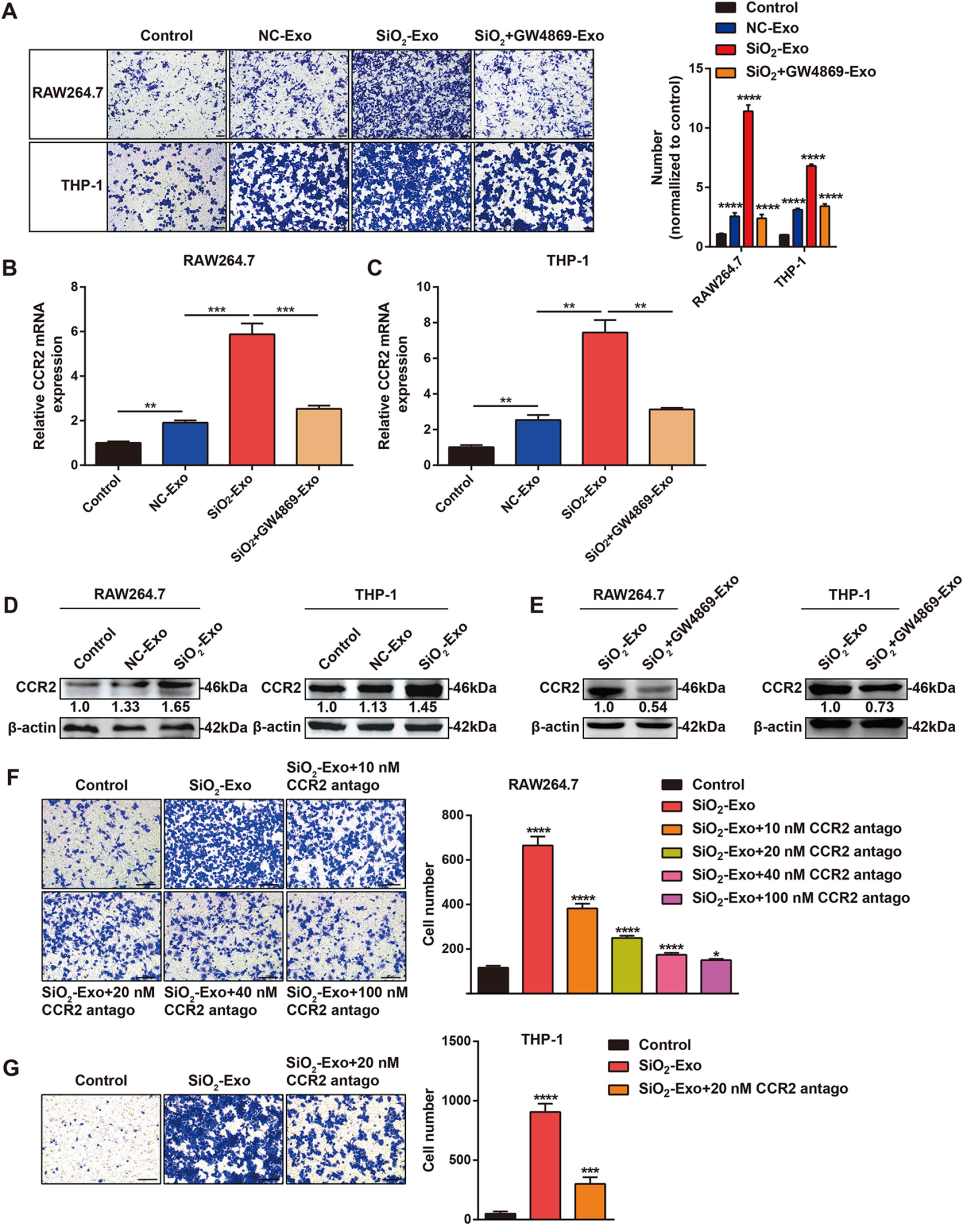
Exosomes derived from SiO_2_-exposed macrophages promote monocyte/macrophage migration through CCR2. **(A)**. Transwell assay of the migration of RAW264.7 macrophages or THP-1 monocytes treated with PBS, NC-Exo, SiO_2_-Exo or SiO_2_ + GW4869-Exo. The scale bar represents 50 μm. **(B-E)**. The expression of CCR2 in RAW264.7 macrophages or THP-1 monocytes treated with PBS, NC-Exo, SiO_2_-Exo or SiO_2_ + GW4869-Exo was analysed by RT–PCR **(B-C)** and western blotting **(D-E)**. **(F-G)**. Transwell assay of the migration of RAW264.7 macrophages or THP-1 monocytes treated with SiO_2_-Exo in the presence of a CCR2 antagonist (10 nM, 20 nM, 40 nM, or 100 nM). The scale bar represents 50 μm. The data are representative of three individual experiments and expressed as the mean ± SEM. The data were analysed by Student’s *t* test or two-way ANOVA. *P < 0.05, **P < 0.01, ***P < 0.001, ****P < 0.0001. *Abbreviations* SiO_2_ = silica dust; NC-Exo = exosomes derived from cells without SiO_2_ exposure; SiO_2_-Exo = exosomes derived from SiO_2_-exposed macrophages; SiO_2_ + GW4869-Exo = exosomes derived from SiO_2_-exposed macrophages treated with GW4869 (10 µM); antago = antagonist

Figure 1A shows that a large number of macrophages infiltrated the lung during the early stage of silicosis. Previous studies have revealed that after SiO_2_ exposure, circulating monocytes migrate into lung tissue to participate in the inflammatory response [5, 23]. Therefore, we further examined whether SiO_2_-Exo regulated monocyte or macrophage migration and recruitment. Transwell assays showed that SiO_2_-Exo significantly promoted the migration of THP-1 monocytes and RAW264.7 macrophages, and the number of migrating cells decreased in the SiO_2_ + GW4869-Exo treatment group (Fig. 4A). CCR2 plays an important role in inflammatory monocyte recruitment. We next evaluated the expression of CCR2 in SiO_2_-Exo-treated monocytes/macrophages. RT‒PCR showed that SiO_2_-Exo upregulated CCR2 expression in THP-1 monocytes and RAW264.7 macrophages, while CCR2 expression was decreased in the SiO_2_ + GW4869-Exo treatment group (Fig. 4B‒C). Western blot analysis also confirmed these results (Fig. 4D-E).

Then, we used a CCR2 antagonist to evaluate the role of CCR2 in the migration of monocytes/macrophages. Transwell assays revealed that the CCR2 antagonist could effectively block the migration of monocytes/macrophages (Fig. 4F-G). These results indicated that SiO_2_-Exo recruited monocytes/macrophages through CCR2 during silica-induced inflammation.

### SiO_2_-Exo promoted the inflammatory response in monocytes/macrophages by regulating the activation of the STAT3/MAPK/NF-κB signalling pathway

**Fig. 5:**
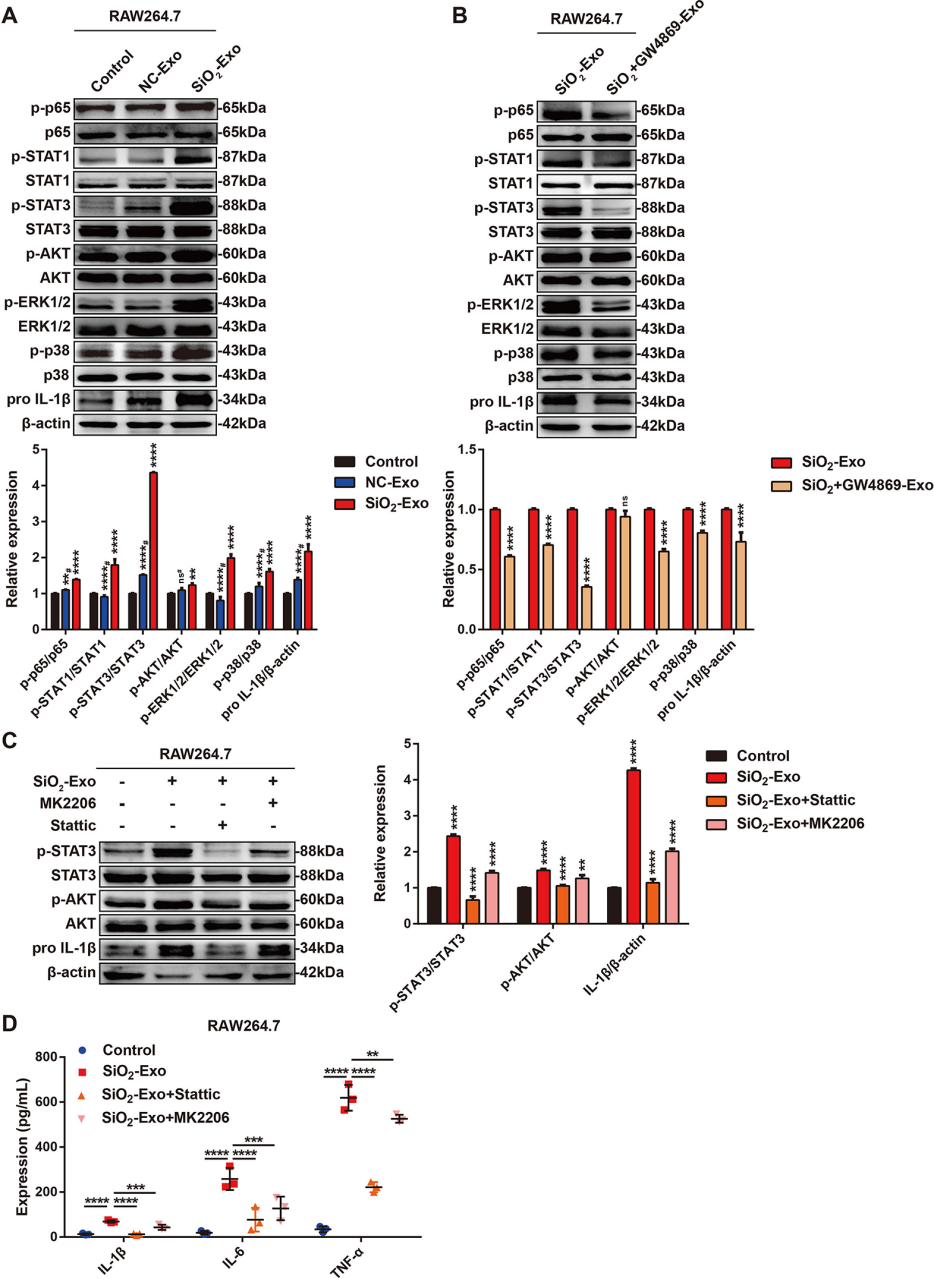
SiO_2_-Exo promotes the inflammatory response by regulating the activation of the STAT3/MAPK (ERK1/2 and p38)/NF-κB signalling pathways. **(A)** Western blot analysis of the expression of pro-IL-1β and the phosphorylation of p65 (NF-κB), STAT1/3, AKT, ERK1/2 and p38 in RAW264.7 macrophages treated with PBS, NC-Exo or SiO_2_-Exo. # indicates that the data were compared between the NC-Exo group and the SiO_2_-Exo group. **(B)** Western blot analysis of the expression of pro-IL-1β and the phosphorylation levels of p65 (NF-κB), STAT1/3, AKT, ERK1/2 and p38 in RAW264.7 macrophages treated with SiO_2_-Exo or SiO_2_ + GW4869-Exo. **(C)** Western blot analysis of the expression of pro-IL-1β and the phosphorylation of STAT3 and AKT in SiO_2_-Exo-induced RAW264.7 macrophages treated with Stattic (5 µM) or MK2206 (10 nM). **(D)** ELISA analysis of the release of IL-1β, IL-6 and TNF-α in the SN of SiO_2_-Exo-induced RAW264.7 macrophages treated with Stattic (5 µM) or MK2206 (10 nM). *n* = 3 each group. The data are representative of three individual experiments and expressed as the mean ± SEM. The data were analysed by Student’s *t* test or two-way ANOVA. *P < 0.05, **P < 0.01, ***P < 0.001, ****P < 0.0001, ns = not significant. *Abbreviations* SiO_2_ = silica dust; SN = cell culture supernatant; NC-Exo = exosomes derived from cells without SiO_2_ exposure; SiO_2_-Exo = exosomes derived from SiO_2_-exposed macrophages; SiO_2_ + GW4869-Exo = exosomes derived from SiO_2_-exposed macrophages treated with GW4869 (10 µM)

To further clarify the mechanism by which exosomes regulate the inflammatory response in monocytes/macrophages, we examined the activation of signalling pathways related to inflammation, such as the nuclear factor kappa-B (NF-κB), signal transducers and activators of transcription (STAT), MAPK (including extracellular signal-regulated kinase 1/2 (ERK1/2) and p38 MAPK), and phosphatidylinositol 3-kinase (PI3K)/protein kinase B (PKB/AKT) (PI3K/AKT) signalling pathways [24–33]. SiO_2_-Exo markedly upregulated the phosphorylation of p65 (NF-κB), STAT1, STAT3, ERK1/2 and p38 in RAW264.7 macrophages, and these changes were accompanied by an increase in pro-IL-1β (Fig. 5A). The phosphorylation levels of p65, STAT1, STAT3, ERK1/2 and p38 were decreased in the SiO_2_ + GW4869-Exo treatment group, while p-AKT expression was not significantly different, and these changes were accompanied by a decrease in pro-IL-1β (Fig. 5B).

In contrast to that in RAW264.7 macrophages, SiO_2_-Exo selectively upregulated the phosphorylation of p65, STAT3, AKT and p38 in THP-1 monocytes, while the phosphorylation of STAT1 and ERK1/2 did not significantly differ. These changes were accompanied by increases in the expression of pro-IL-1β and CD68 (Figure S3A). The phosphorylation of p65, STAT3, ERK1/2 and p38 was downregulated in the SiO_2_ + GW4869-Exo treatment group, while the expression of p-STAT1 and p-AKT was not significantly different. These changes were accompanied by a decrease in the expression of pro-IL-1β (Figure S3B). These results indicated that SiO_2_-Exo promoted the inflammatory response of monocytes/macrophages mainly by inducing the activation of the STAT3/MAPK (ERK1/2 and p38)/NF-κB signalling pathways. The signalling cascades mediated by SiO_2_-Exo in mouse leukaemic macrophages (RAW264.7 cells) and human monocytes (THP-1 cells) showed some differences.

STAT3 and AKT play dual roles in inflammation. To further verify the role of STAT3- and AKT-related signalling in SiO_2_-Exo-induced monocyte/macrophage activation, we treated RAW264.7 macrophages and THP-1 monocytes with SiO_2_-Exo in the presence of a STAT3 inhibitor (Stattic, 5 µM) or an AKT inhibitor (MK2206, 10 nM) and analysed the inflammatory response. The addition of Stattic and MK2206 significantly inhibited the phosphorylation of STAT3 and AKT induced by SiO_2_-Exo, and these changes were accompanied by a decrease in pro-IL-1β (Fig. 5C, Figure S3C). ELISA analysis revealed that Stattic and MK2206 could significantly attenuate the release of TNF-α, IL-6 and IL-1β induced by SiO_2_-Exo (Fig. 5D). Notably, Stattic had a stronger inhibitory effect than MK2206. These results suggested that STAT3 and AKT promoted the transcription of inflammatory cytokines during the SiO_2_-Exo-induced inflammatory activation of monocytes/macrophages.

### HMGB3 protein expression was increased in macrophage-derived exosomes after SiO_2_ exposure

**Fig. 6:**
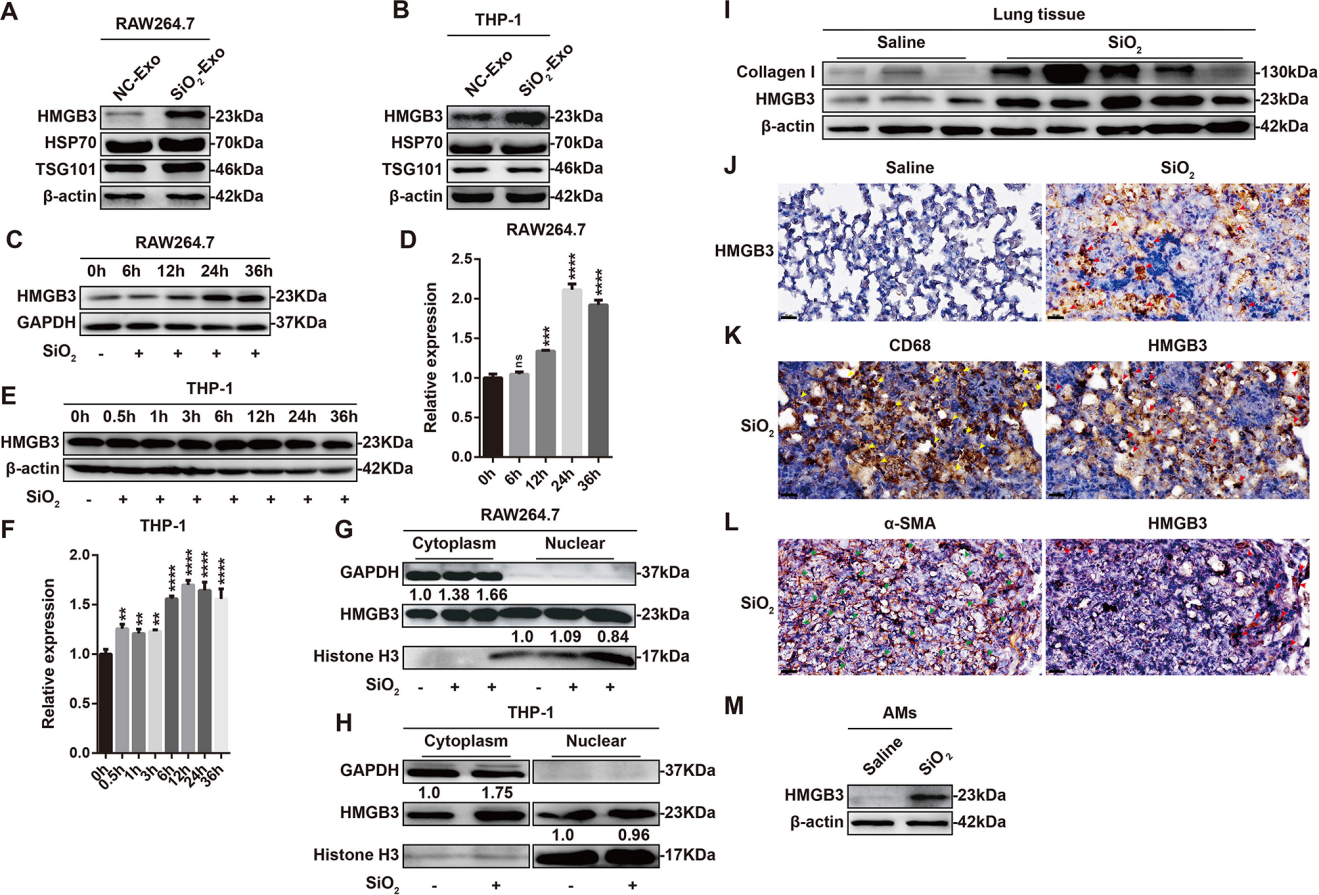
HMGB3 protein expression is increased in SiO_2_-Exo and SiO_2_-exposed macrophages. **(A-B)**. Western blot analysis of the expression of HMGB3, HSP70, TSG101 and β-actin in exosomes derived from RAW264.7 macrophages or THP-1 macrophages with or without SiO_2_ exposure. **(C-F)**. Western blot analysis of HMGB3 expression in RAW264.7 macrophages or THP-1 macrophages exposed to SiO_2_ for different times. **(G-H)**. Western blot analysis of HMGB3 protein expression in the cytoplasm and nucleus of RAW264.7 macrophages or THP-1 macrophages exposed to SiO_2_. **(I)**. Western blot analysis of the expression of collagen type I and HMGB3 in the lung tissue of normal mice and mice with silicosis. *n* = 3 mice in the normal group (saline) and *n* = 5 mice in the silicosis group (SiO_2_). **(J)**. Representative image showing immunohistochemical staining of HMGB3 (red arrowheads) in the lung tissue of normal mice and mice with silicosis. The scale bar represents 20 μm. **(K)**. Representative image showing immunohistochemical staining of CD68 (a macrophage-related marker, yellow arrowheads) and HMGB3 (red arrowheads) in the lung tissue of mice with silicosis. The scale bar represents 20 μm. **(L)**. Representative image showing immunohistochemical staining of α-SMA (a myofibroblast-related marker, green arrowheads) and HMGB3 (red arrowheads) in the lung tissue of mice with silicosis. The scale bar represents 20 μm. **(M)**. Representative western blot showing HMGB3 expression in the alveolar macrophages (AMs) of normal mice and mice with silicosis. *n* = 15 mice per group. The data are representative of three individual experiments and expressed as the mean ± SEM. The data were analysed by Student’s *t* test. *P < 0.05, **P < 0.01, ***P < 0.001, ****P < 0.0001, ns = not significant. *Abbreviations* SiO_2_ = silica dust; AMs = alveolar macrophages; NC-Exo = exosomes derived from cells without SiO_2_ exposure; SiO_2_-Exo = exosomes derived from SiO_2_-exposed macrophages

A previous report revealed that HMGB3 expression was upregulated in exosomes derived from SiO_2_-exposed RAW264.7 cells [22]. Therefore, we subsequently examined the role of HMGB3 in the inflammatory response of monocytes/macrophages induced by SiO_2_-Exo. First, we examined HMGB3 expression in exosomes derived from macrophages with or without SiO_2_ exposure, and the results indicated that HMGB3 expression in SiO_2_-Exo was higher than that in NC-Exo (Fig. 6A-B). We further measured the expression of HMGB1 and HMGB2 in macrophage-derived exosomes. The results showed no difference between NC-Exo and SiO_2_-Exo (Figure S4A-B). Considering that the variation in the contents of exosomes is typically consistent with their source cells, we next examined the protein expression of HMGB3 in macrophages after SiO_2_ exposure for different times. The results suggested that HMGB3 protein expression was increased in RAW264.7 macrophages and THP-1 macrophages after SiO_2_ exposure (Fig. 6C-F). HMGB1 and HMGB2 can be transferred from the nucleus to the cytoplasm in response to stress or elevated ROS production [18, 34]. Previous studies revealed that ROS levels are increased in macrophages after the phagocytosis of silica particles [35, 36]. Therefore, we extracted cytoplasmic and nuclear proteins from SiO_2_-exposed macrophages and examined the protein expression of HMGB3. The results suggested that HMGB3 protein levels were increased in the cytoplasm after SiO_2_ exposure, but there was no significant difference in HMGB3 protein levels in the nucleus (Fig. 6G-H). These results indicated that HMGB3 could be transferred from the nucleus to the cytoplasm in response to SiO_2_-induced stress, where the HMGB3 protein was then packaged into exosomes and secreted into the extracellular environment. We next investigated the expression of the HMGB3 protein in mice with silicosis. High expression of collagen I indicated that we had successfully constructed a silicosis mouse model and that the protein expression of HMGB3 was increased in the lung tissues of mice with silicosis (Fig. 6I). Immunohistochemical staining revealed that the HMGB3 protein was expressed at low levels in the lung tissue of normal mice treated with saline but was increased in mice with silicosis (red arrowheads) (Fig. 6J); this protein was mainly expressed in infiltrating macrophages (CD68, yellow arrowheads) (Fig. 6K) rather than in myofibroblasts (α-SMA, green arrowheads) (Fig. 6L). Consistent with these results, the protein expression of HMGB3 was upregulated in alveolar macrophages from mice with silicosis (Fig. 6M).

### HMGB3 deficiency attenuated SiO_2_-Exo-induced inflammatory activation and the recruitment of monocytes/macrophages in vitro and in vivo

**Fig. 7:**
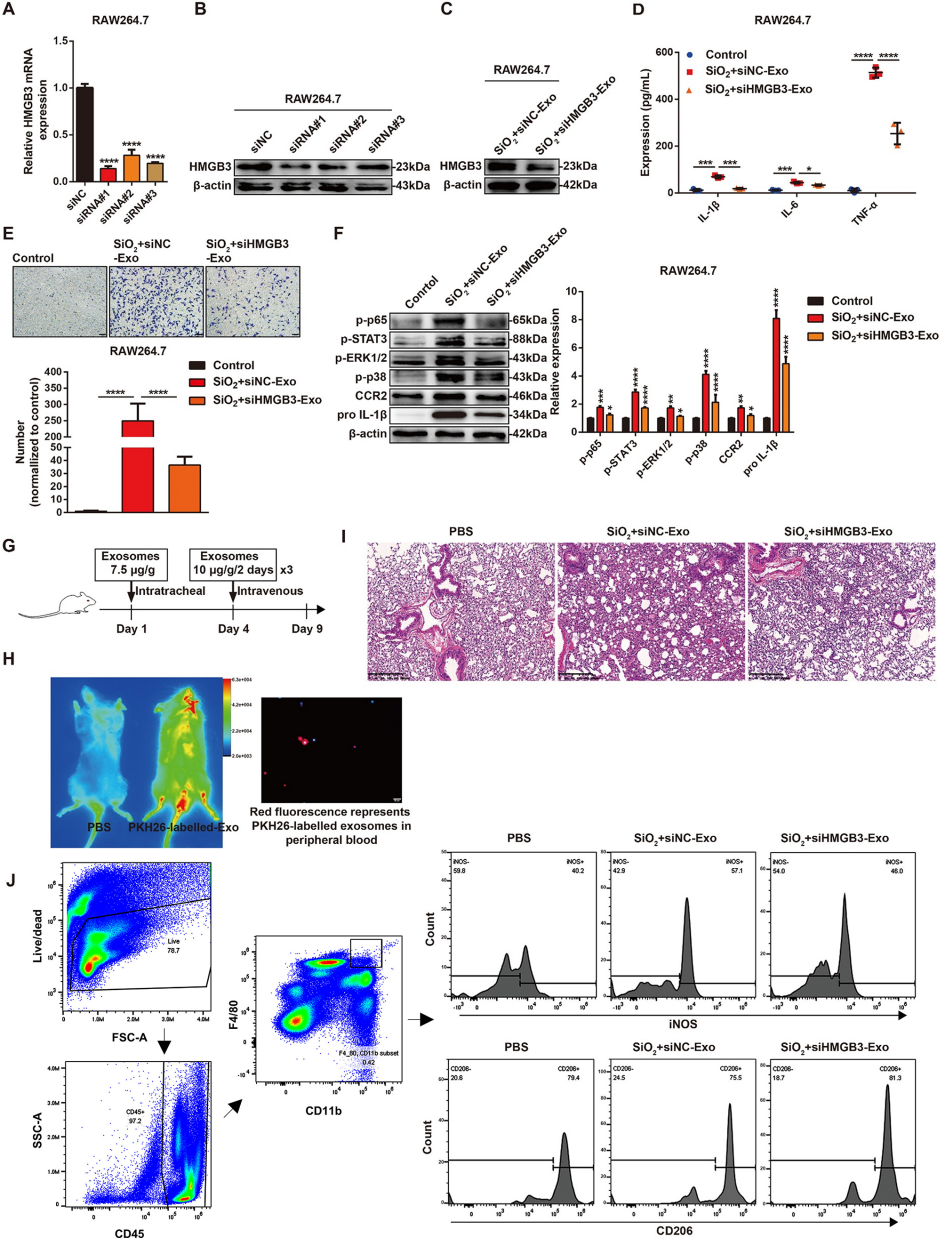
Knocking down HMGB3 attenuates the inflammatory activation and migration induced by SiO_2_-Exo in macrophages. **(A-B)**. RT–PCR and western blot analysis of HMGB3-silenced RAW264.7 macrophages. **(C)**. Western blot analysis of HMGB3 expression in exosomes derived from macrophages transfected with siNC or siHMGB3. **(D)**. ELISA analysis of the expression of IL-1β, IL-6 and TNF-α in the SN of RAW264.7 macrophages treated with PBS, SiO_2_ + siNC-Exo, or SiO_2_ + siHMGB3-Exo. *n* = 3 each group. **(E)**. Transwell assay of the migration of RAW264.7 macrophages treated with PBS, SiO_2_ + siNC-Exo, or SiO_2_ + siHMGB3-Exo. The scale bar represents 50 μm. **(F)**. Western blot analysis of the expression of p-p65, p-STAT3, p-ERK1/2, p-p38, CCR2 and pro-IL-1β in RAW264.7 macrophages treated with PBS, SiO_2_ + siNC-Exo, or SiO_2_ + siHMGB3-Exo. **(G)**. Flow chart showing the process of exosome administration in mice. **(H)**. The distribution of PKH26-labelled exosomes in mice was observed by an in vivo Xtreme II system, and PKH26-labelled exosomes in peripheral blood were observed by fluorescence microscopy. The scale bar represents 20 μm. **(I)**. HE staining of lung tissue from mice treated with PBS, SiO_2_ + siNC-Exo, or SiO_2_ + siHMGB3-Exo. *n* = 5 mice per group. **(J)**. Flow cytometry gating strategy for CD11b^+^/F4/80^+^ subsets. Flow cytometric analysis of the proportions of iNOS^+^ or CD206^+^ macrophages in the lung tissue of mice treated with PBS, SiO_2_ + siNC-Exo, or SiO_2_ + siHMGB3-Exo. *n* = 5 mice per group. The data are representative of three individual experiments and expressed as the mean ± SEM. The data were analysed by Student’s *t* test or two-way ANOVA. *P < 0.05, **P < 0.01, ***P < 0.001, ****P < 0.0001. *Abbreviations* SiO_2_ = silica dust; SN = cell culture supernatant; SiO_2_ + siNC-Exo = exosomes derived from SiO_2_-exposed macrophages transfected with siNC; SiO_2_ + siHMGB3-Exo = exosomes derived from SiO_2_-exposed macrophages transfected with siHMGB3

To investigate the role of HMGB3 in SiO_2_-Exo-induced inflammatory activation and the recruitment of monocytes/macrophages, we constructed three siRNAs or shRNAs to knock down HMGB3. The knockdown efficiency of the siRNAs in RAW264.7 macrophages or shRNAs in THP-1 macrophages was assessed by RT‒PCR and western blot analysis, respectively (Fig. 7A-B, Figure S5A-B), and the results showed that siHMGB3#1 and shHMGB3#1 had the greatest gene silencing effects. These sequences were selected for subsequent experiments.

We transfected RAW264.7 macrophages with siRNAs and THP-1 macrophages with shRNAs, and then isolated exosomes from the SN after 36 h of silica exposure. Exosomes were obtained from siNC/siHMGB3-transfected RAW264.7 macrophages (designated as SiO_2_ + siNC-Exo or SiO_2_ + siHMGB3-Exo) and shNC/shHMGB3-transfected THP-1 macrophages (designated as SiO_2_ + shNC-Exo or SiO_2_ + shHMGB3-Exo). HMGB3 expression in the exosomes was measured by western blot analysis, and the results demonstrated that HMGB3 expression in SiO_2_ + siHMGB3-Exo and SiO_2_ + shHMGB3-Exo was lower than that in SiO_2_ + siNC-Exo and SiO_2_ + shNC-Exo (Fig. 7C, Figure S5C). We next evaluated the role of HMGB3 in SiO_2_-Exo-induced inflammatory responses by treating RAW264.7 macrophages or THP-1 monocytes with exosomes and measuring the release of inflammatory cytokines and cell migration by ELISA and transwell assays, respectively. The results showed a marked increase in the expression of IL-1β, IL-6 and TNF-α in the SiO_2_ + siNC-Exo-treated group compared with the control group, and knockdown of exosomal HMGB3 partially reversed this effect (Fig. 7D). SiO_2_ + siNC/shNC-Exo significantly promoted the migration of RAW264.7 macrophages (Fig. 7E) and THP-1 monocytes (Figure S5D), and the migration of these cells decreased after exosomal HMGB3 was knocked down.

Exosomes were then cocultured with RAW264.7 macrophages and THP-1 monocytes, and the activation of signalling pathways was evaluated by western blot analysis. SiO_2_ + siNC-Exo upregulated the expression of p-p65, p-STAT3, p-ERK1/2, p-p38, CCR2 and pro-IL-1β in RAW264.7 macrophages. When exosomal HMGB3 was knocked down, the expression of p-p65, p-STAT3, p-ERK1/2, p-p38, CCR2 and pro-IL-1β decreased (Fig. 7F). In THP-1 monocytes, the expression of p-p65, p-STAT3, p-p38, CCR2 and pro-IL-1β was significantly upregulated in the SiO_2_ + shNC-Exo treatment group, while p-ERK1/2 expression was downregulated. When exosomal HMGB3 was knocked down, the expression of p-p65, p-STAT3, p-p38, CCR2 and pro-IL-1β was downregulated (Figure S5E).

We next investigated the role of exosomal HMGB3 in vivo by constructing a mouse model of pulmonary inflammation induced by exosomes through intratracheal injection and tail vein injection (Fig. 7G). PKH26-labelled exosomes were administered to the mice by tail vein injection, and the distribution of the exosomes was observed 20 h later by an in vivo Xtreme II system, which showed that PKH26-labelled exosomes or cells that took up PKH26-labelled exosomes were distributed in lung tissue (Fig. 7H). PKH26 dye was also found in nucleated cells in the peripheral blood by fluorescence microscopy (Fig. 7H). HE staining revealed alveolar structure destruction and interstitial hyperplasia in the SiO_2_ + siNC-Exo treatment group compared with the control group (PBS), and lung tissue damage was attenuated in the SiO_2_ + siHMGB3-Exo treatment group (Fig. 7I). The lungs were also harvested for flow cytometric analysis after the mice were sacrificed. The results demonstrated that the proportion of iNOS^+^ macrophages was higher in mice treated with SiO_2_ + siNC-Exo than in the PBS-treated group. Knocking down exosomal HMGB3 partially decreased the proportion of iNOS^+^ macrophages (Fig. 7J). However, the proportions of CD206^+^ macrophages were not significantly different (Fig. 7J).

### HMGB3-enriched exosomes promoted inflammatory activation and monocyte/macrophage migration in vitro

**Fig. 8:**
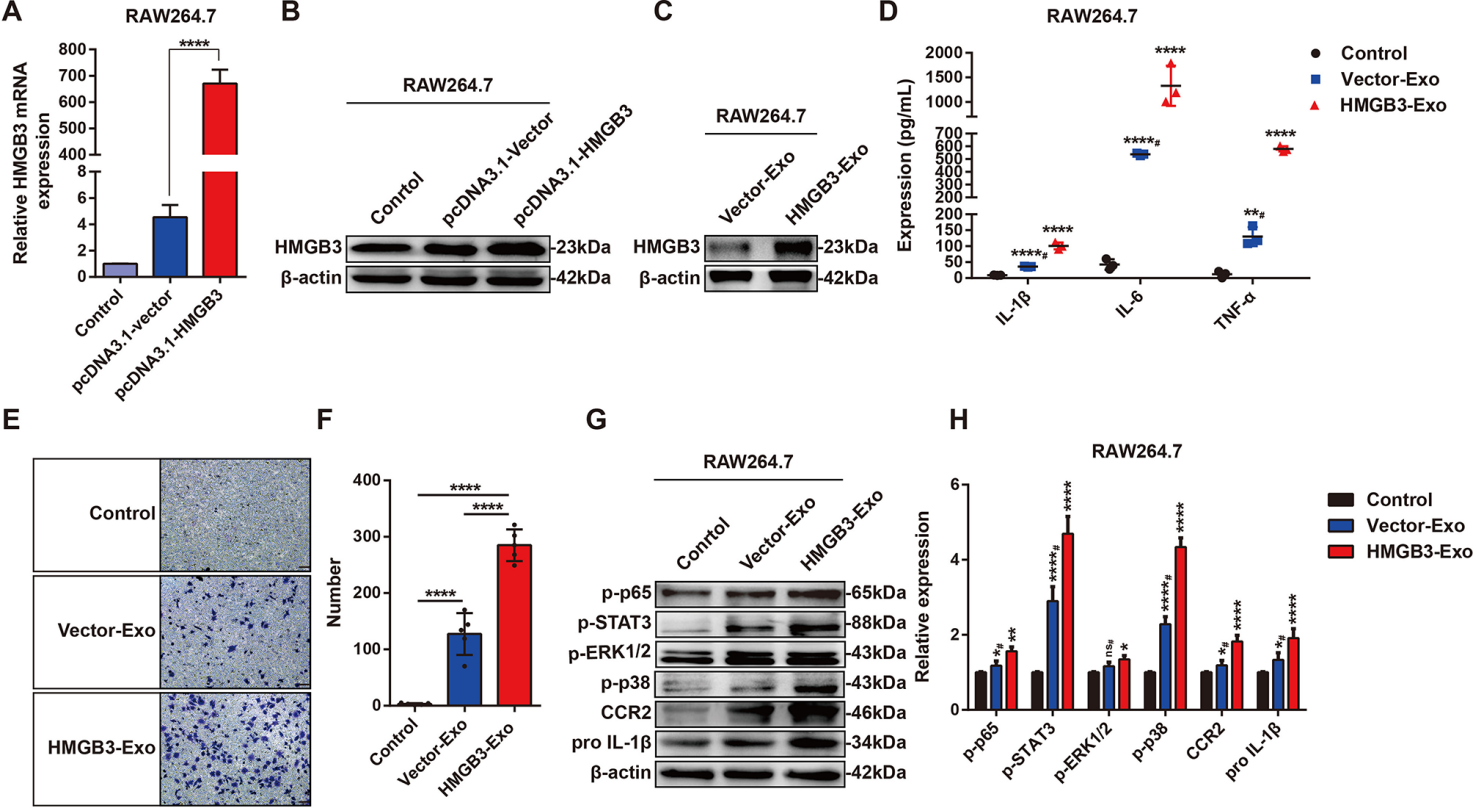
Overexpression of exosomal HMGB3 enhances the release of proinflammatory cytokines and the migration of macrophages. **(A-B)**. RT–PCR and western blot analysis of the transfection efficiency of the pcDNA3.1-HMGB3 plasmid in RAW264.7 cells. **(C)**. Western blot analysis of HMGB3 expression in exosomes derived from macrophages transfected with pcDNA3.1-vector or pcDNA3.1-HMGB3. **(D)**. ELISA analysis of the levels of IL-1β, IL-6 and TNF-α in the SN of RAW264.7 macrophages treated with PBS, Vector-Exo, or HMGB3-Exo. *n* = 3 each group. **(E-F)**. Transwell assay of the migration of RAW264.7 macrophages treated with PBS, Vector-Exo, or HMGB3-Exo. The scale bar represents 50 μm. **(G-H)**. Western blot analysis of the expression of p-p65, p-STAT3, p-ERK1/2, p-p38, CCR2 and pro IL-1β in RAW264.7 macrophages treated with PBS, Vector-Exo, or HMGB3-Exo. # indicates that the data were compared between the Vector-Exo group and the HMGB3-Exo group. The data are representative of three individual experiments and expressed as the mean ± SEM. The data were analysed by Student’s *t* test. *P < 0.05, **P < 0.01, ***P < 0.001, ****P < 0.0001, ns = not significant. *Abbreviations* SN = cell culture supernatant; Vector-Exo = exosomes derived from macrophages transfected with the vector plasmid; HMGB3-Exo = exosomes derived from macrophages transfected with the HMGB3 plasmid

We next constructed a pcDNA3.1 (+)-HMGB3 plasmid, and the transfection efficiency of pcDNA3.1-HMGB3 in RAW264.7 cells was evaluated by RT‒PCR and western blot analysis (Fig. 8A-B). RAW264.7 macrophages were transfected with the plasmid, and the SN was collected for exosome isolation, resulting in Vector-Exo derived from macrophages transfected with pcDNA3.1-vector and HMGB3-Exo derived from macrophages transfected with pcDNA3.1-HMGB3. HMGB3 expression in these exosomes was assessed by western blot analysis, and the results showed that HMGB3 expression in HMGB3-Exo was upregulated compared with that in Vector-Exo (Fig. 8C). These exosomes were then cocultured with M0 RAW264.7 macrophages, after which macrophage activation and migration were measured. ELISA analysis revealed significant increases in the release of IL-1β, IL-6 and TNF-α from HMGB3-Exo-treated macrophages (Fig. 8D). Transwell assays showed that HMGB3-Exo significantly promoted RAW264.7 macrophage migration (Fig. 8E-F). We then examined the activation of signalling pathways in macrophages by western blot analysis, and the results demonstrated that HMGB3-Exo upregulated the expression of p-p65, p-STAT3, p-ERK1/2, p-p38, CCR2 and pro-IL-1β (Fig. 8G-H). These results revealed that HMGB3 was involved in SiO_2_-Exo-induced inflammatory activation and recruitment of macrophages by regulating activation of the STAT3/MAPK (ERK1/2 and p38)/NF-κB/CCR2 signalling pathways.

## Discussion

Silicosis is an irreversible and fatal lung disease characterized by chronic inflammation and fibrosis [23, 37], but the inflammatory mediators involved have not been fully elucidated. Macrophages are key effector cells in silicosis that exhibit significant heterogeneity in different stages of silicosis. Zhao et al. reported that the proportion of M1 macrophages began to increase in alveolar lavage fluid on Day 7 after silica exposure and peaked on Day 14; moreover, IL-1β and TNF-α expression peaked on Day 28, and the proportion of M2 macrophages began to increase 42 days postexposure [6]. Consistent with the findings of previous reports, we found increased macrophage infiltration in lung tissues with a predominance of the M1 subtype in a 28-day silicosis mouse model. Interestingly, we observed that exosome secretion by macrophages was significantly increased after SiO_2_ exposure. Moreover, the excessive secretion of exosomes in pathological conditions contributes to disease progression, including sepsis, idiopathic pulmonary fibrosis (IPF) and asthma [11, 13, 38, 39], and exosomes are widely involved in many diseases, such as tubulointerstitial inflammation, glioma, and sepsis, by regulating macrophage polarization [40–42]. However, the role of exosomes in silica-induced inflammation has not yet been elucidated. Our study revealed that exosomes derived from silica-exposed macrophages played a proinflammatory role in silica-induced inflammation by promoting M1 polarization and the recruitment of monocytes/macrophages.

A previous study reported that the proportion of Ly6C^hi^/CCR2^+^ monocytes was increased in lung tissue 3 days after silica exposure [23]. The recruitment of circulating monocytes to inflammatory sites is regulated by chemokines, and the most critical chemokines are monocyte chemokines (MCPs), which regulate cell migration by activating homologous chemokine receptors, including CCR2 [43]. CCR2^+^CX3CR1^+^ monocytes are preferentially recruited and acquire proinflammatory properties during glomerulonephritis [44]. CCR2-deficient mice exhibit significantly decreased monocyte recruitment during peritonitis, autoimmune encephalitis, tuberculosis, and atherosclerosis [45]. Our findings showed that exosomes derived from silica-exposed macrophages recruited circulating monocytes through CCR2 in silica-induced inflammation.

HMGB3 belongs to the HMGB family and has an 80% homologous amino acid sequence and a similar structure to those of HMGB1 and HMGB2 [46]. A previous study revealed that the binding of HMGB1, HMGB2 and HMGB3 to nucleic acids could activate toll-like receptor 3 (TLR3)-, TLR7- and TLR9-mediated innate immune responses, which were accompanied by activation of the interferon regulatory factor 3 (IRF3) and NF-κB signalling pathways and the induction of inflammatory cytokine transcription [19]. As DAMPs, HMGB1 and HMGB2 also can induce cytokine transcription by binding to TLR2, TLR4 and receptor of advanced glycation endproducts (RAGE), triggering a cascade of inflammatory signalling pathways [19, 47]. HMGB1 can also bind to C-X-C motif chemokine ligand 12 (CXCL12) to form a heterocomplex, which induces monocyte recruitment via C-X-C motif chemokine receptor 4 (CXCR4) [48]. However, the function and regulatory mechanisms of HMGB3, which has a similar structure to HMGB1 and HMGB2, in inflammation have not been clarified, and whether HMGB3 can directly bind to TLRs to induce an inflammatory response has not yet been determined. Our study suggested that HMGB3 was a key effector in the SiO_2_-Exo-induced inflammatory response and that exosomal HMGB3 could widely induce inflammatory signalling cascades, including the STAT3, MAPK and NF-κB signalling pathways. However, the specific underlying mechanism still needs to be further explored.

Previous studies have revealed that macrophage-derived exosomes have profibrotic effects on silicosis by promoting myofibroblast differentiation and epithelial–mesenchymal transition [22, 49, 50]. Our results indicated that exosomes secreted by macrophages exposed to silica had a strong proinflammatory effect and promoted inflammatory monocyte recruitment and infiltration. Blocking exosome secretion in vivo can attenuate pulmonary inflammation and fibrosis in mice with silicosis [14]. These results suggest that macrophage-derived exosomes are key risk factors for silicosis. Secreted exosomes deliver their contents to recipient cells mainly through endocytosis, membrane fusion and receptor‒ligand-mediated interactions [9, 51, 52]. Endocytosis is the most common pathway through which exosomes are taken up into endosomal compartments, where TLR3, TLR7, TLR8, and TLR9 are present [53], and are correlated with inflammation and fibrosis [54, 55]. Therefore, further exploration of how exosomes trigger signalling cascades within recipient cells and for the identification of potential blocking sites may provide new insights into silicosis therapy.

In summary, the present study indicated that SiO_2_-Exo was proinflammatory factor in silica-induced inflammation that promoted M1 polarization and the recruitment of monocytes/macrophages, and these processes were regulated by activation of the STAT3/MAPK (ERK1/2 and p38)/NF-κB/CCR2 signalling pathways via exosomal HMGB3. These findings might lead to the identification of therapeutic targets for early treatment of silicosis-related inflammation.

## Conclusions

In the present study, we found that silica stimulation enhanced exosome secretion by macrophages and that the secreted exosomes regulated silica-induced inflammation by promoting M1 polarization and the recruitment of monocytes/macrophages. Notably, HMGB3 expression was increased in these exosomes, and HMGB3 acted as a key effector of SiO_2_-Exo-induced inflammatory activation and the recruitment of monocytes/macrophages by regulating activation of the STAT3/MAPK/NF-κB/CCR2 signalling pathways. Our work provides new insights into the chronic inflammation associated with silicosis.

### Electronic supplementary material

Below is the link to the electronic supplementary material.

Supplementary Material 1

## Data Availability

The datasets supporting the conclusions of this article are included within the article and can be retrieved from the corresponding author upon reasonable request.

## Abbreviations

SiO_2_: Silica
MCP-1: Monocyte chemotactic protein-1
TNF-α: Tumor necrosis factor-alpha
IL-1β: Interleukin-1 beta
HMGB3: High mobility group box 3
DAMP: Damage-associated molecular pattern
SN: Cell culture supernatant
TSG101: Tumor susceptibility gene 101
HSP70: Heat shock protein 70
TEM: Transmission electron microscopy
NTA: Nanoparticle tracking analysis
PDPN: Podoplanin
GAPDH: Glyceraldehyde3-phosphate dehydrogenase
ELISA: Enzyme-linked immunosorbent assay
RT-PCR: Reverse transcription-polymerase chain reaction
AMs: Alveolar macrophages
BALF: Bronchoalveolar lavage fluid
Exos: Exosomes
NC-Exo: Exosomes derived from macrophages without SiO_2_ exposure
SiO_2_-Exo: Exosomes derived from SiO_2_-exposed macrophages
SiO2 + GW4869-Exo: Exosomes derived from SiO_2_-exposed macrophages treated with GW4869
CCR2: C-C motif chemokine receptor 2
STAT: Signal transducers and activators of transcription
NF-κB: Nuclear factor kappa-B
PI3K/AKT: Phosphatidylinositol 3-kinase (PI3K)/protein kinase B (PKB/AKT)
MAPK: Mitogen-activated protein kinase
ERK1/2: Extracellular signal-regulated kinase 1/2
HMGBs: HMGB family
ROS: Reactive oxygen species
TLRs: Toll-like receptors
RAGE: Receptor of advanced glycation endproducts
