# Supplementary material for: Macrophage-derived exosomal HMGB3 regulates silica-induced pulmonary inflammation by promoting M1 macrophage polarization and recruitment
Source: Part Fibre Toxicol. 2024 Mar 7;21:12. doi: 10.1186/s12989-024-00568-8 (PMC10918916; doi:10.1186/s12989-024-00568-8)

**Supplementary materials**

**Supplementary Table**

Table S1 The primer sequences used for qPCR analysis

| gene | primer sequence | |
| --- | --- | --- |
| CCR2-mouse | FORWARD | 5’-GACTACGATGATGGTGAGCCTTGTC-3’ |
|  | REVERSE | 5’-GTAATGTGAGCAGGAAGAGCAGGTC-3’ |
| CCR2-human | FORWARD | 5’-TGGTGGCTGTGTTTGCTTCTGTC-3’ |
|  | REVERSE | 5’-GCCCTATGCCTCTTCTTCTCGTTTC-3’ |
| HMGB3-mouse | FORWARD | 5’-TCAAATCCACAAACCCTGGCATCTC-3’ |
|  | REVERSE | 5’-CATCTTCCTCCTCTTCCTCCTCCTC-3’ |
| HMGB3-human | FORWARD | 5’-CACCGTCTGGATTCTTCCTGTTCTG-3’ |
|  | REVERSE | 5’-TCCTCCTCCTCCTCCTCCTCTTC-3’ |
| IL-1β-mouse | FORWARD | 5’-TGCCACCTTTTGACAGTGATG-3’ |
|  | REVERSE | 5’-AAGGTCCACGGGAAAGACAC-3’ |
| IL-6-mouse | FORWARD | 5’-AGAGGATACCACTCCCAACAGACC-3’ |
|  | REVERSE | 5’-AGCCACTCCTTCTGTGACTCCAG-3’ |
| TNF-α-mouse | FORWARD | 5’-GCGACGTGGAACTGGCAGAAG-3’ |
|  | REVERSE | 5’-GTGGTTTGTGAGTGTGAGGGTCTG-3’ |
| IL-10-mouse | FORWARD | 5’-CTGCTATGCTGCCTGCTCTTACTG-3’ |
|  | REVERSE | 5’-AGCCGCATCCTGAGGGTCTTC-3’ |
| GAPDH-mouse | FORWARD | 5’-TCACCATCTTCCAGGAGCGAGAC-3’ |
|  | REVERSE | 5’-TGAGCCCTTCCACAATGCCAAAG-3’ |
| β-actin-mouse | FORWARD | 5’-GCGGGCGACGATGCT-3’ |
|  | REVERSE | 5’-TCATCTTTTCACGGTTGGCCT-3’ |
| IL-1β- human | FORWARD | 5’-CAGAAGTACCTGAGCTCGCC-3’ |
|  | REVERSE | 5’-AGATTCGTAGCTGGATGCCG-3’ |
| IL-6- human | FORWARD | 5’-GAACTCCTTCTCCACAAGCG-3’ |
|  | REVERSE | 5’-GCCTCTTTGCTGCTTTCACA-3’ |
| TNF-α- human | FORWARD | 5’-AAGCCTGTAGCCCATGTTGT-3’ |
|  | REVERSE | 5’-GAGGTACAGGCCCTCTGATG-3’ |
| IL-10- human | FORWARD | 5’-CCTGCCTAACATGCTTCGAG-3’ |
|  | REVERSE | 5’-AGTTCACATGCGCCTTGATG-3’ |
| GAPDH- human | FORWARD | 5’-GGCACCGTCAAGGCTGAGAAC-3’ |
|  | REVERSE | 5’-TGCAGGAGGCATTGCTGATGATC-3’ |
| β-actin- human | FORWARD | 5’-GGGCATGGGTCAGAAGGATT-3’ |
|  | REVERSE | 5’-GAGGCGTACAGGGATAGCAC-3’ |

**Supplementary Figures**

To explore the role of SiO_2_-Exo in silica-induced inflammation, we blocked exosome secretion by SiO_2_-exposed macrophages with GW4869 (10 μM) as a control to detect SiO_2_-Exo-induced inflammatory activation in monocytes/macrophages. The results showed that GW4869 effectively inhibited exosome secretion of SiO_2_-exposed macrophages.


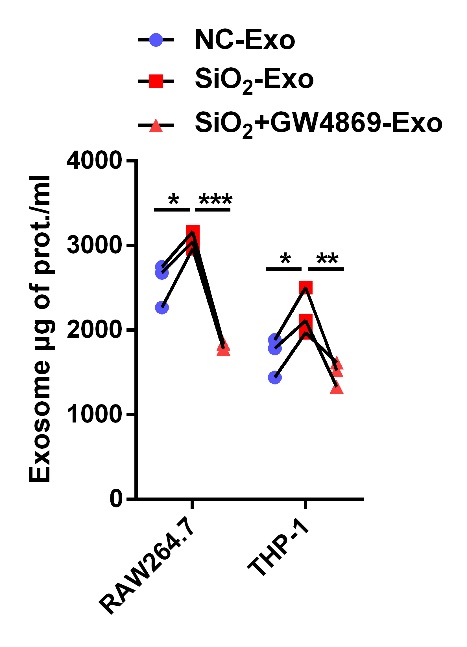


**Figure S1** The secretion of exosomes by macrophages is decreased by GW4869 treatment. Micro-BCA assay analysis of the total protein content of exosomes derived from RAW264.7 macrophages and THP-1 macrophages treated with or without GW4869 (10 μM). n = 3 per group. The data were analysed by two-way ANOVA. **P* < 0.05, ***P* < 0.01, ****P* < 0.001. *Abbreviations* SiO_2_ = silica dust; NC-Exo = exosomes derived from cells without SiO_2_ exposure; SiO_2_-Exo = exosomes derived from SiO_2_-exposed macrophages; SiO_2_ + GW4869-Exo = exosomes derived from SiO_2_-exposed macrophage treated with GW4869

The growth pattern of THP-1 monocytes changed from a suspending to adhesive growth after SiO_2_-Exo treatment (Figure 3B), which prompted us to detect whether SiO_2_-Exo could induce macrophage differentiation. Immunofluorescence analysis of THP-1 cells revealed that SiO_2_-Exo upregulated CD68 expression, promoted the differentiation of monocyte into macrophage (Figure S2A). Moreover, the CCK-8 assay showed that SiO_2_-Exo had no effect on the proliferation of RAW264.7 macrophages (Figure S2B).


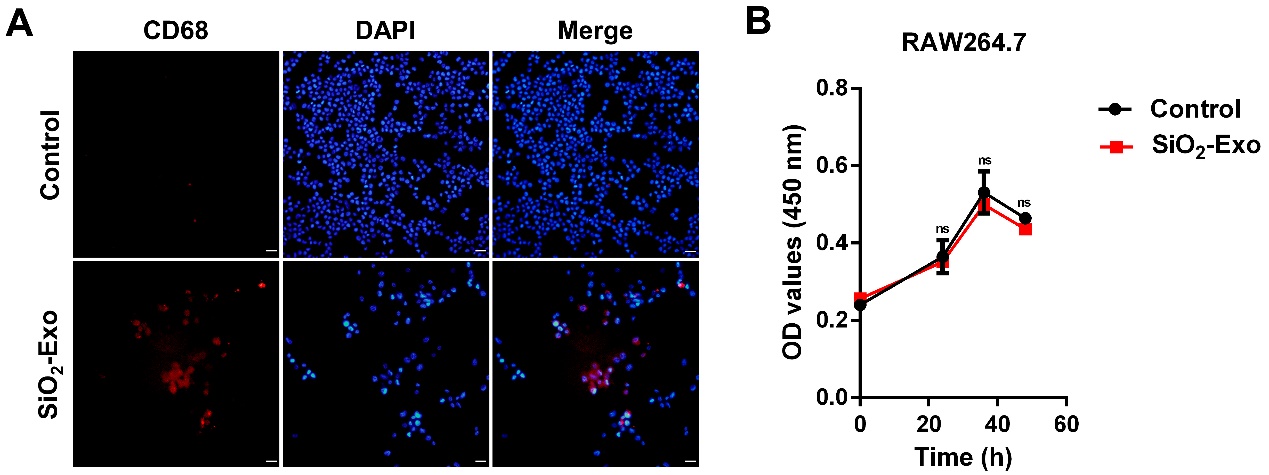


**Figure S2** SiO_2_-Exo promotes the differentiation of monocytes into macrophages in THP-1 cells, whereas it has no effect on the proliferation of RAW264.7 macrophages. **(A)**. Immunofluorescence analysis of CD68 expression in THP-1 monocytes after PBS or SiO2-Exo treatment. The scale bar represents 50 μm. **(B)**. CCK-8 assay analysis of the proliferation of RAW264.7 macrophages treated with PBS or SiO_2_-Exo. The data are representative of three individual experiments and expressed as the mean ± SEM. The data were analyzed by two-way ANOVA. **P* < 0.05, ns = not significant. *Abbreviations* SiO_2_ = silica dust; SiO_2_-Exo = exosomes derived from SiO_2_-exposed macrophages

To clarify the mechanism by which exosomes regulate the inflammatory response in monocytes, we examined the activation of signalling pathways related to inflammation, such as NF-κB, STAT, MAPK (including ERK1/2 and p38), and PI3K/AKT signalling pathways. SiO_2_-Exo selectively upregulated the phosphorylation of p65, STAT3, AKT and p38 in THP-1 monocytes, while the phosphorylation of STAT1 and ERK1/2 did not significantly differ; these changes were accompanied by increases in the expression of pro-IL-1β and CD68 (Figure S3A). The phosphorylation of p65, STAT3, ERK1/2 and p38 was downregulated in the SiO_2_ + GW4869-Exo treatment group, while the expression of p-STAT1 and p-AKT was not significantly different; these changes were accompanied by a decrease in the expression of pro-IL-1β (Figure S3B). The treatment of STAT3 inhibitor (Stattic, 5 μM) or AKT inhibitor (MK2206, 10 nM) significantly blunted the phosphorylation of STAT3 and AKT induced by SiO_2_-Exo, and these changes were accompanied by a decrease in pro-IL-1β (Figure S3C). The results indicated that SiO_2_-Exo promoted the inflammatory response of monocytes mainly by inducing the activation of the STAT3/MAPK /NF-κB signalling pathways.


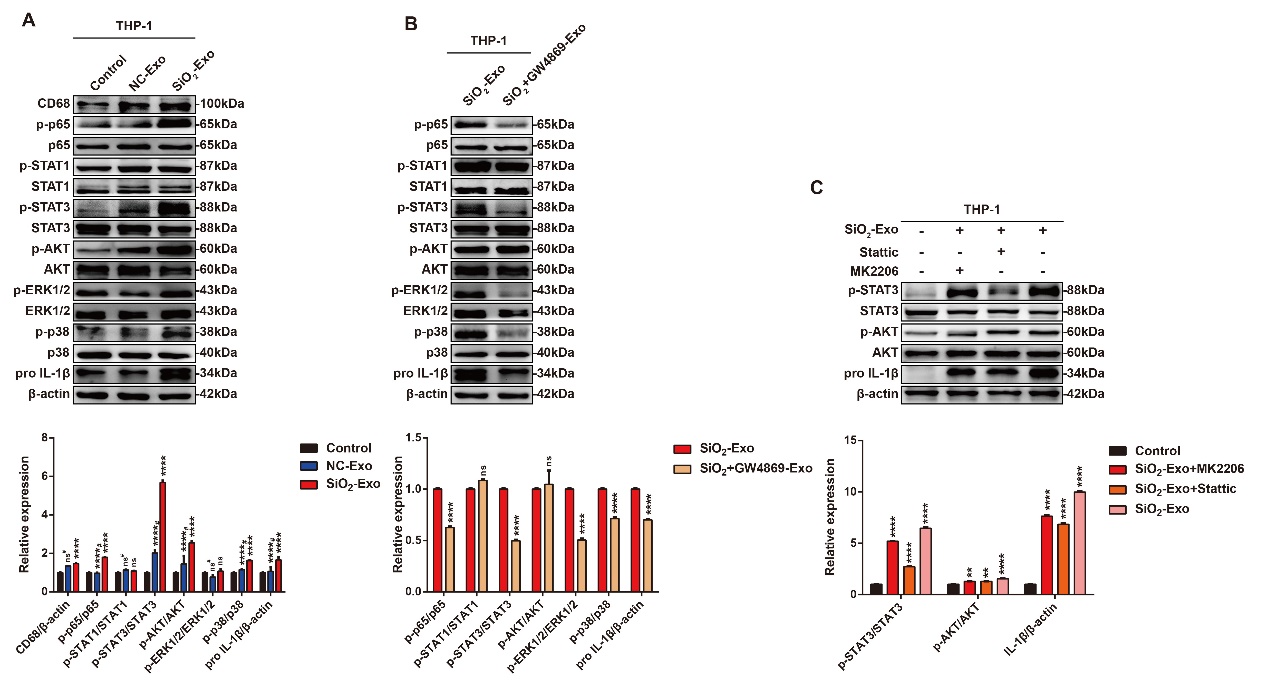


**Figure S3** SiO_2_-Exo promotes the inflammatory response by regulating the activation of the STAT3/MAPK/NF-κB signalling pathways. **(A)**. Western blot analysis of the expression of CD68 and pro-IL-1β and the phosphorylation of p65 (NF-κB), STAT1/3, AKT, ERK1/2 and p38 in THP-1 monocytes treated with PBS, NC-Exo or SiO_2_-Exo. # indicates that the data were compared between the NC-Exo group and the SiO_2_-Exo group. **(B)**. Western blot analysis of the expression of pro-IL-1β and the phosphorylation levels of p65 (NF-κB), STAT1/3, AKT, ERK1/2 and p38 in THP-1 monocytes treated with SiO_2_-Exo or SiO_2_ + GW4869-Exo. **(C)**. Western blot analysis of the expression of pro-IL-1β and the phosphorylation of STAT3 and AKT in SiO_2_-Exo-induced THP-1 monocytes treated with Stattic (5 μM) or MK2206 (10 nM). The data are representative data from three individual experiments and expressed as the mean ± SEM. The data were analyzed by two-way ANOVA. **P* < 0.05, ***P* < 0.01, ****P* < 0.001, *****P* < 0.0001, ns = not significant. *Abbreviations* SiO_2_ = silica dust; NC-Exo = exosomes derived from cells without SiO_2_ exposure; SiO_2_-Exo = exosomes derived from SiO_2_-exposed macrophages; SiO_2_ + GW4869-Exo = exosomes derived from SiO_2_-exposed macrophages treated with GW4869 (10 μM)

Figure 6A-B showed that HMGB3 expression was upregulated in SiO_2_-Exo compared to NC-Exo. HMGB3 belongs to the HMGB family which has an 80% homologous amino acid sequence and a similar structure to those of HMGB1 and HMGB2 (1). Previous studies have revealed that HMGB1 and HMGB2 are involved in innate immunity and inflammation by inducing cytokine production (2-4). Therefore, we further measured the expression of HMGB1 and HMGB2 in macrophage-derived exosomes and there was no difference between NC-Exo and SiO_2_-Exo (Figure S4A-B).

**
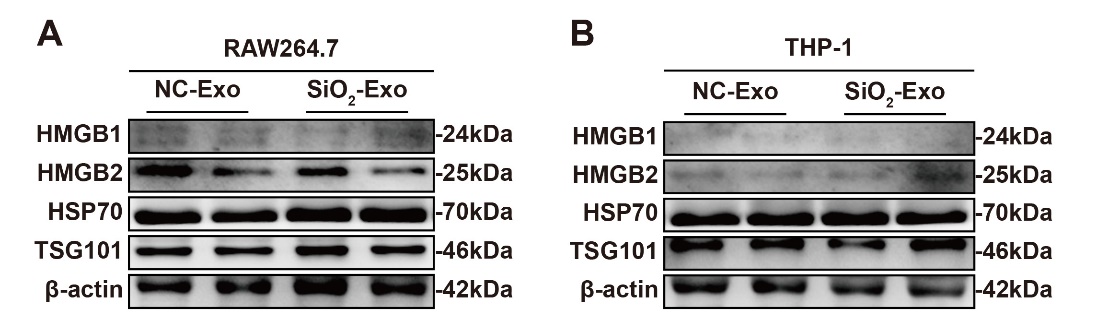
**

**Figure S4** The expression of HMGB1 and HMGB2 is not significantly different between NC-Exo and SiO_2_-Exo. **(A-B)**. Western blot analysis of the expression of HMGB1, HMGB2, HSP70, TSG101 and β-actin in exosomes derived from RAW264.7 macrophages or THP-1 macrophages with or without SiO_2_ exposure. *Abbreviation* NC-Exo = exosomes derived from cells without SiO_2_ exposure; SiO_2_-Exo = exosomes derived from SiO_2_-exposed macrophages.

To investigate the role of HMGB3 in SiO_2_-Exo-induced inflammatory activation and the recruitment of monocytes, we transfected THP-1 macrophages with shRNAs and detected the knockdown efficiency by RT‒PCR and western blot analysis (Figure S5A-C). We next evaluated the inflammatory responses in THP-1 monocytes exposed to SiO_2_ + shNC-Exo or SiO_2_ + shHMGB3-Exo, by measuring cell migration and analysing the expression of inflammatory cytokine and the activation status of involved signalling pathways. When exosomal HMGB3 was knocked down, the migration of THP-1 monocytes decreased (Figure S5D), and the expression of p-p65, p-STAT3, p-p38, CCR2 and pro-IL-1β was downregulated (Figure S5E).


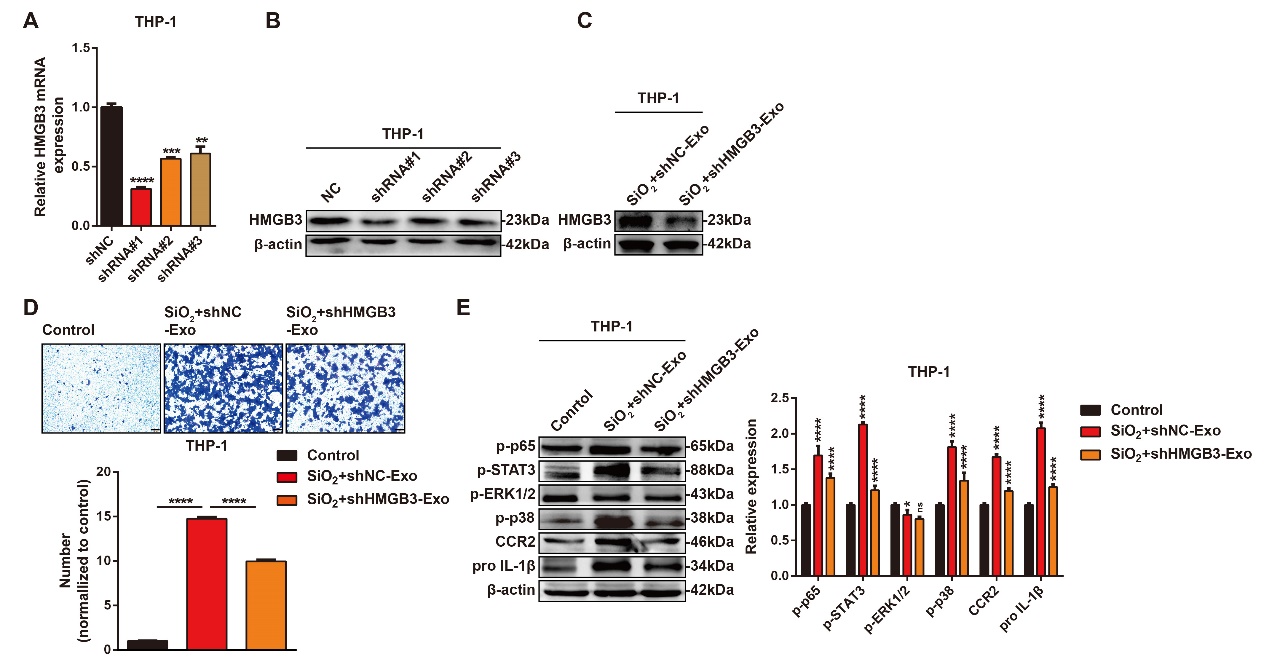


**Figure S5** Knocking down HMGB3 attenuates the inflammatory activation and migration induced by SiO_2_-Exo in monocytes. **(A-B)**. RT‒PCR and western blot analysis of HMGB3-silenced THP-1 macrophages. **(C)**. Western blot analysis of HMGB3 expression in exosomes derived from macrophages transfected with shNC or shHMGB3. **(D)**. Transwell assay of the migration of THP-1 monocytes treated with PBS, SiO_2_ + shNC-Exo, or SiO_2_ + shHMGB3-Exo. The scale bar represents 50 μm. **(E)**. Western blot analysis of the expression of p-p65, p-STAT3, p-ERK1/2, p-p38, CCR2 and pro-IL-1β in THP-1 monocytes treated with PBS, SiO_2_ + shNC-Exo, or SiO_2_ + shHMGB3-Exo. The data are representative of three individual experiments and expressed as the mean ± SEM. The data were analysed by Student's *t* test or two-way ANOVA. **P* < 0.05, ***P* < 0.01, ****P* < 0.001, *****P* < 0.0001. *Abbreviations* SiO_2_ = silica dust; SiO_2_ + shNC-Exo = exosomes derived from SiO_2_-exposed macrophages transfected with shNC; SiO_2_ + shHMGB3-Exo = exosomes derived from SiO_2_-exposed macrophages transfected with shHMGB3

**References**

1. Vaccari T, Beltrame M, Ferrari S, Bianchi ME. Hmg4, a new member of the Hmg1/2 gene family. Genomics. 1998;49(2):247-52.

2. Tian J, Avalos AM, Mao SY, Chen B, Senthil K, Wu H, et al. Toll-like receptor 9-dependent activation by DNA-containing immune complexes is mediated by HMGB1 and RAGE. Nat Immunol. 2007;8(5):487-96.

3. Scaffidi P, Misteli T, Bianchi ME. Release of chromatin protein HMGB1 by necrotic cells triggers inflammation. Nature. 2002;418(6894):191-5.

4. Yanai H, Ban T, Wang Z, Choi MK, Kawamura T, Negishi H, et al. HMGB proteins function as universal sentinels for nucleic-acid-mediated innate immune responses. Nature. 2009;462(7269):99-103.

The original western blot images are listed below:

**Figure 1**


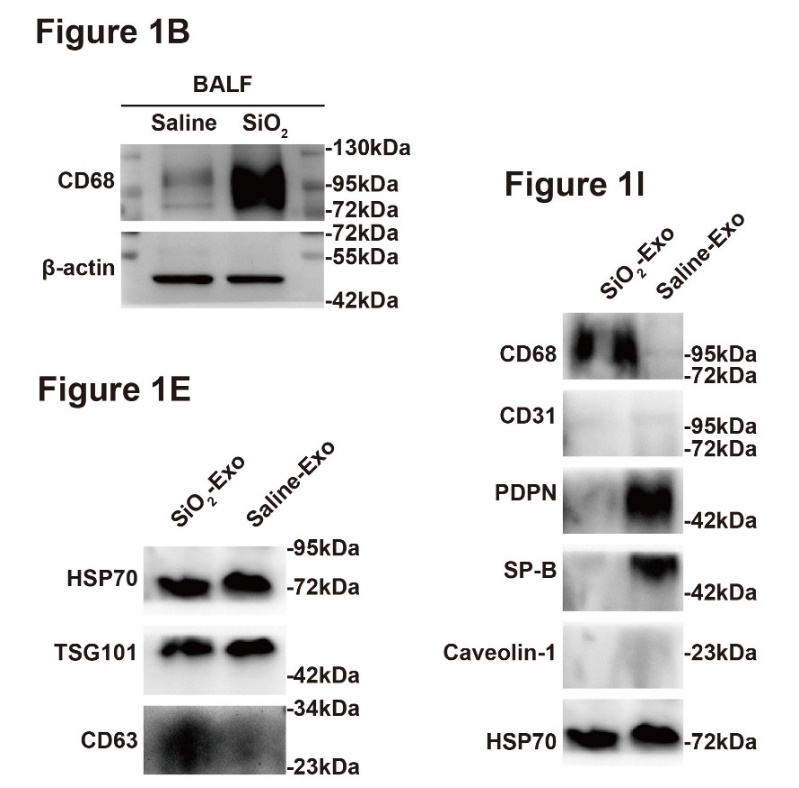


**Figure 2**


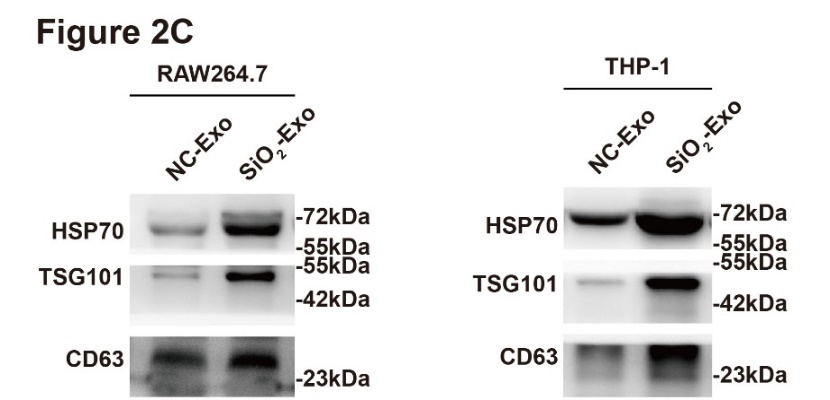


**Figure 4**


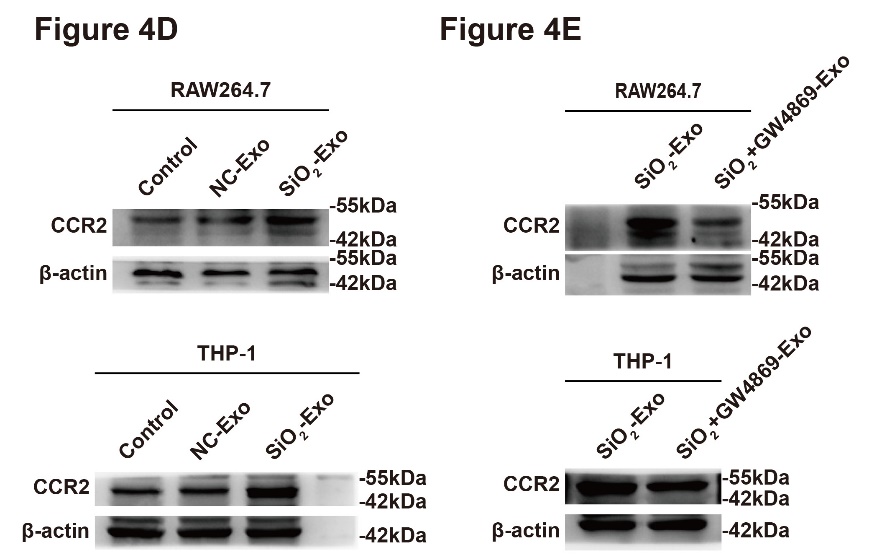


**Figure 5**


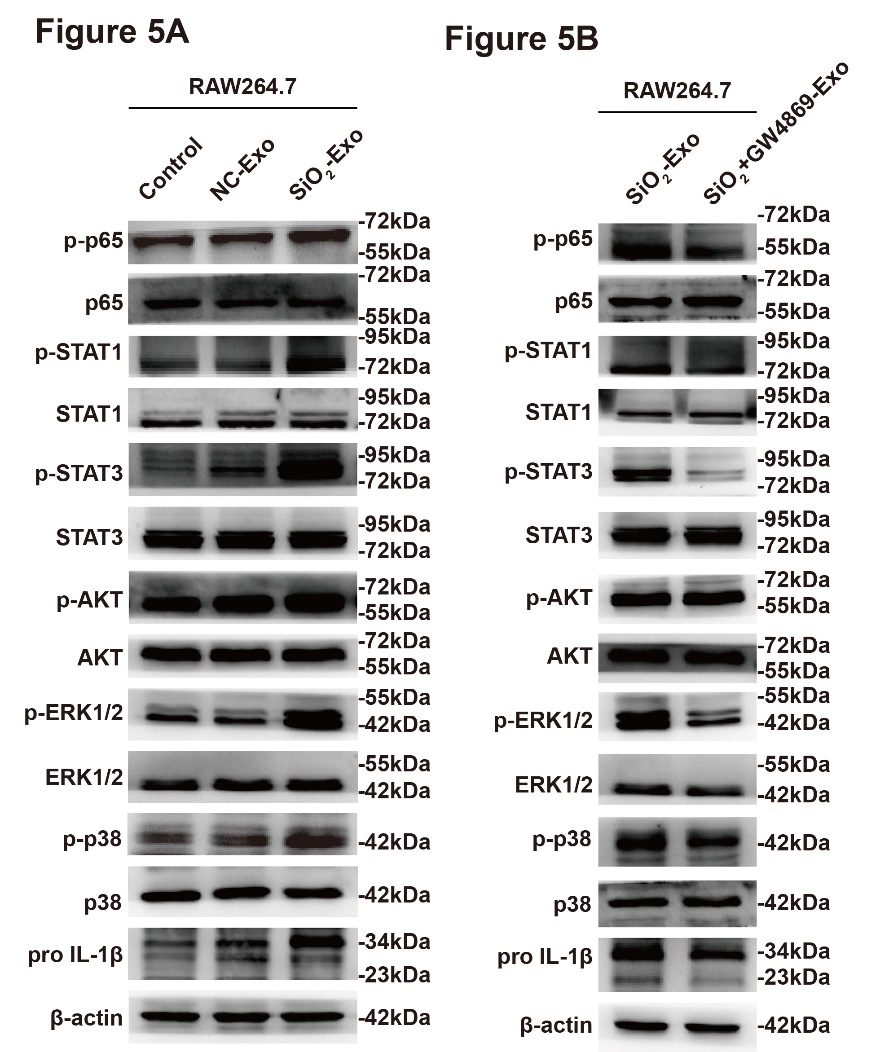

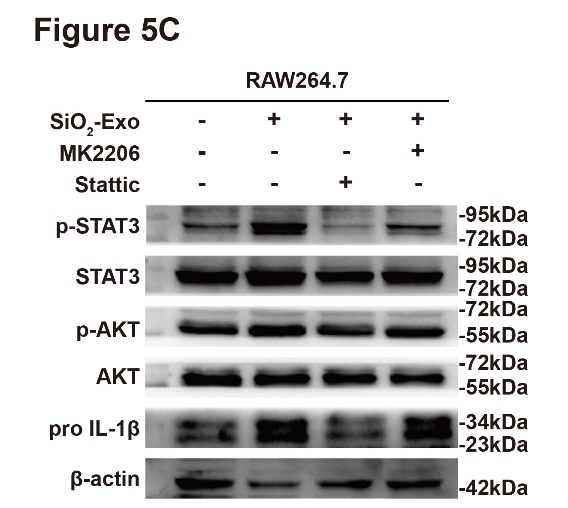


**Figure 6**


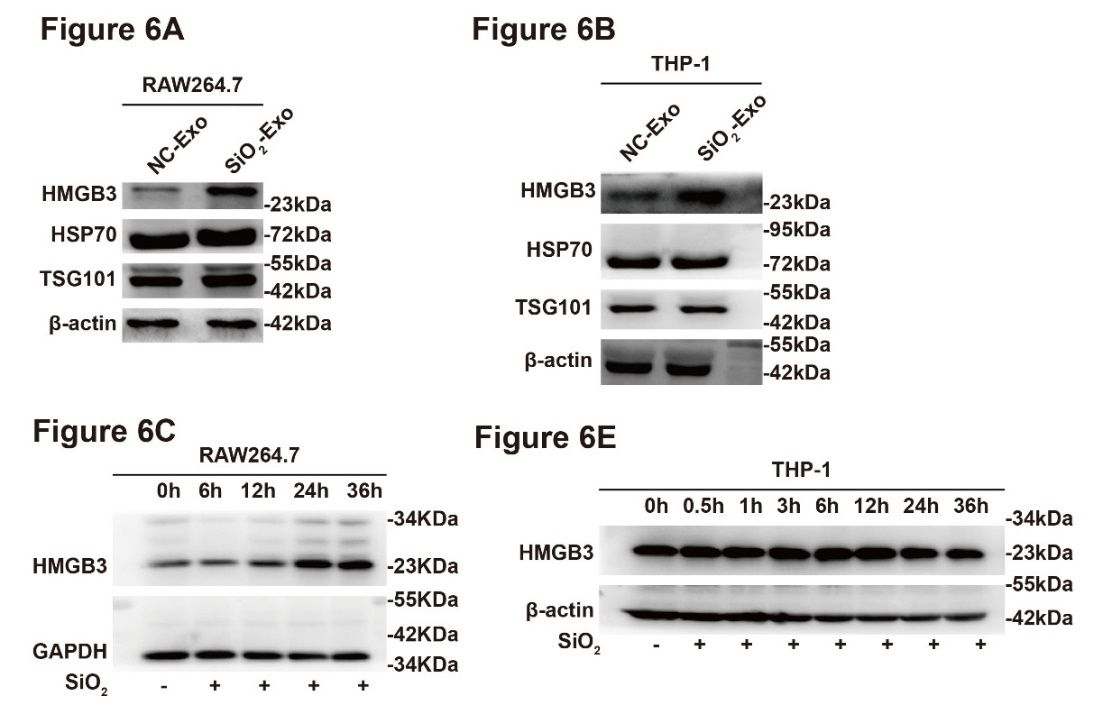


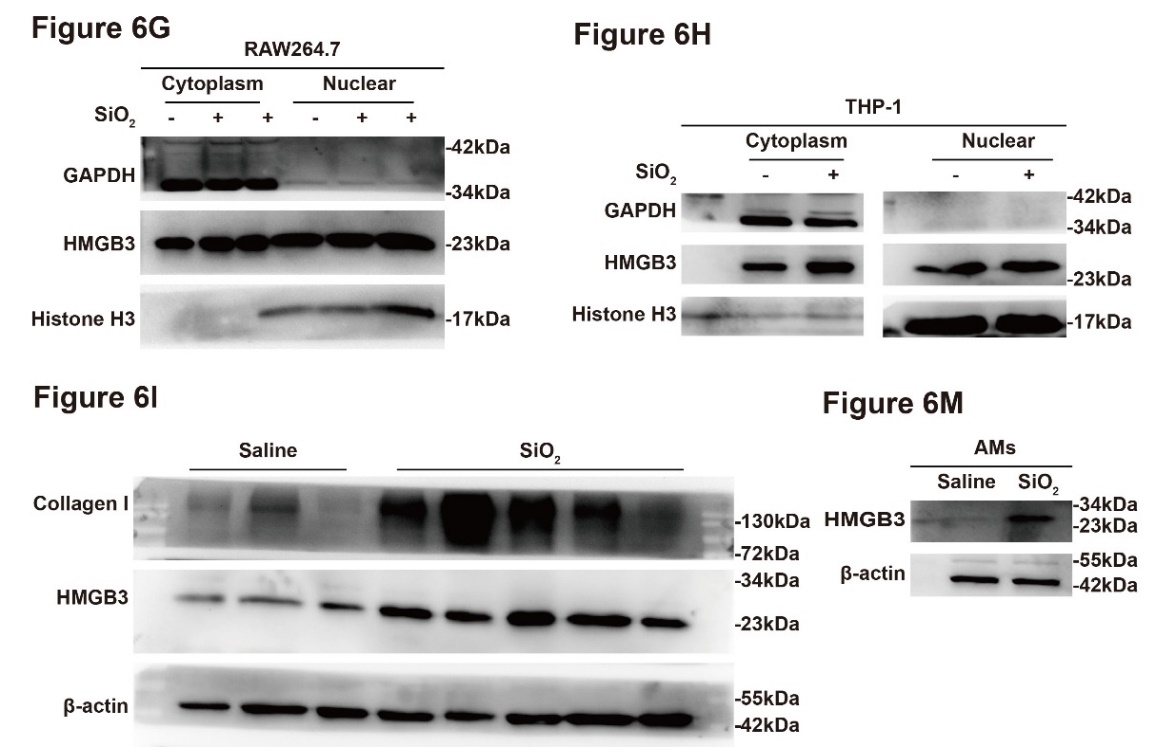


**Figure 7**


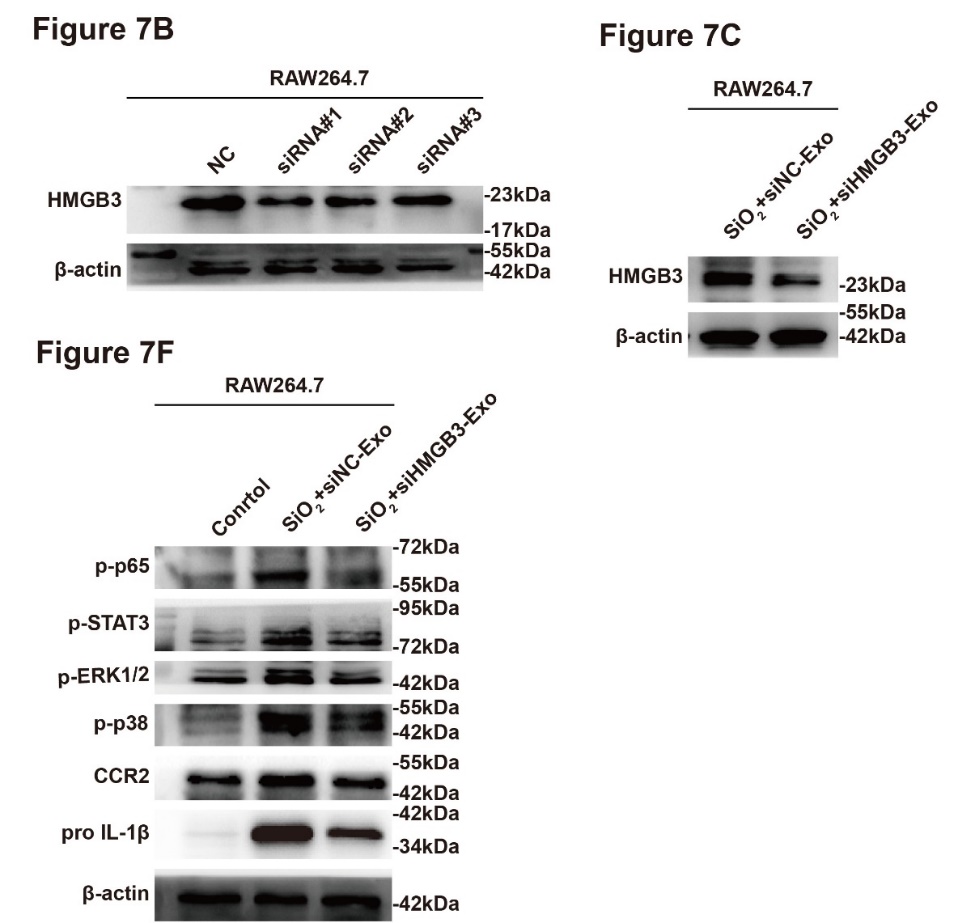


**Figure 8**


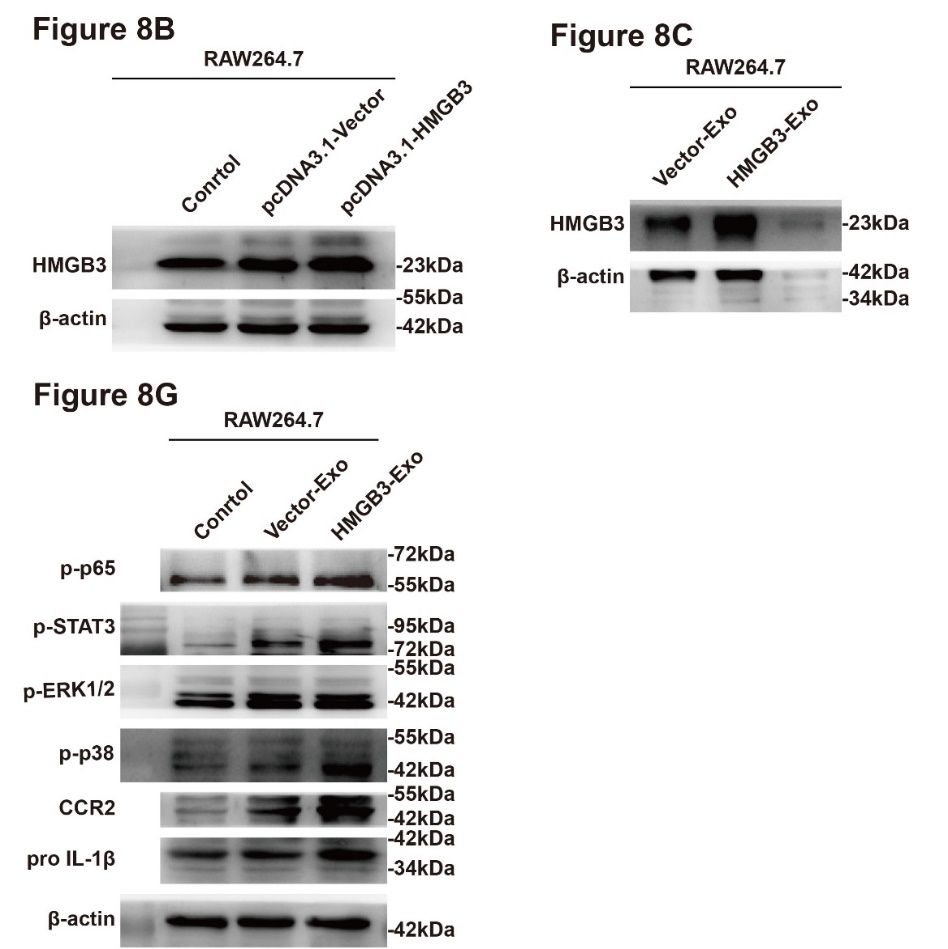


**Figure S3**


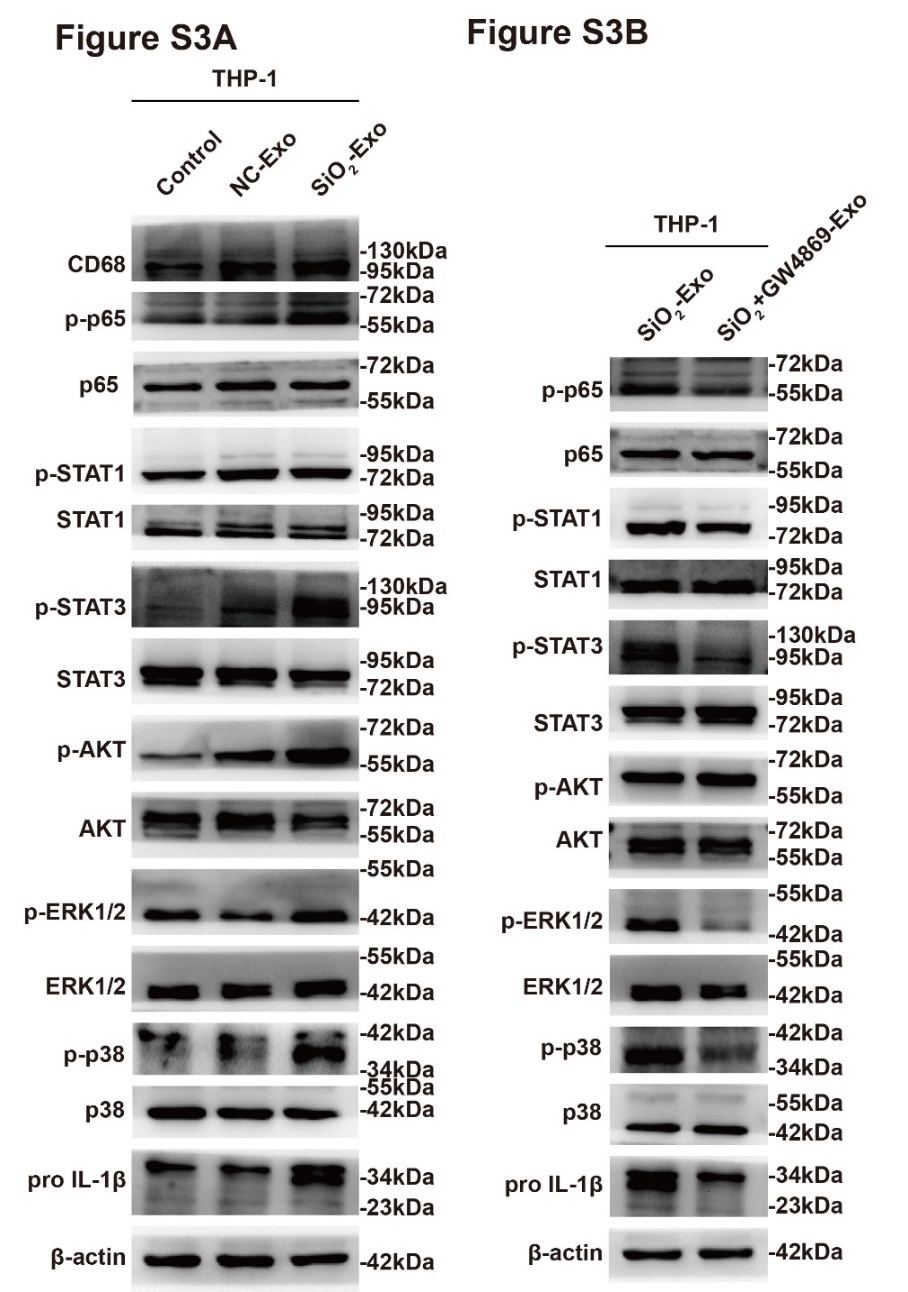


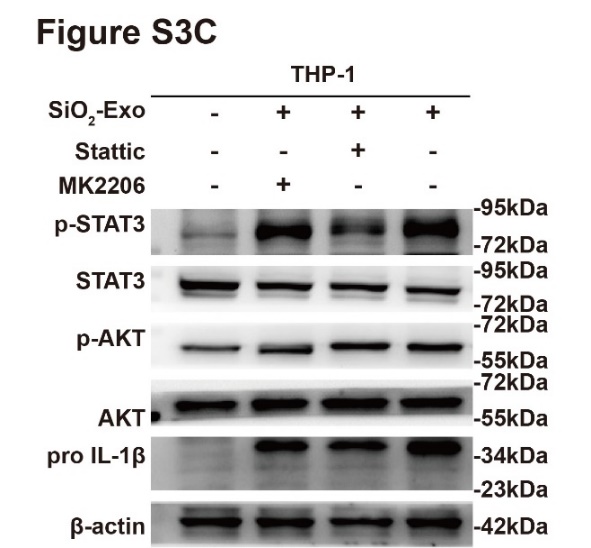


**Figure S4**

**
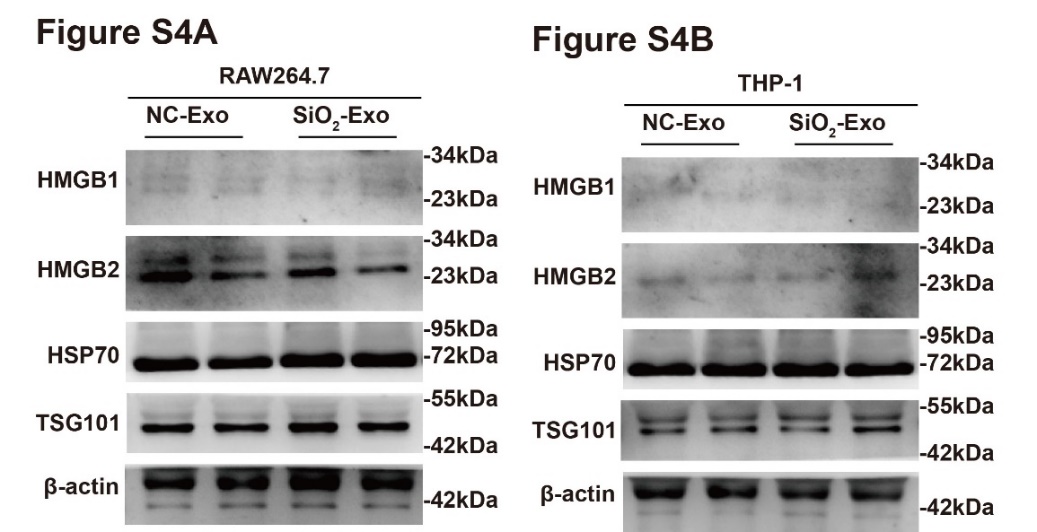
**

**Figure S5**


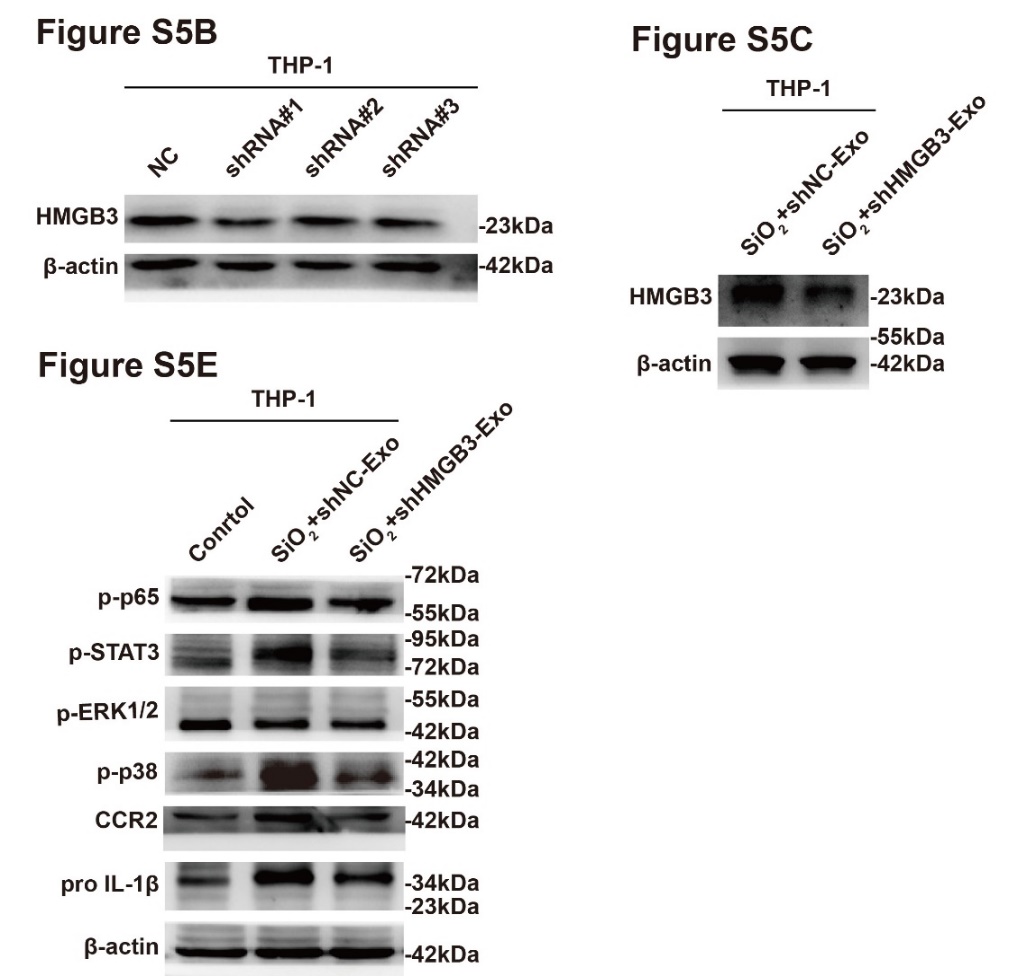

Supplement: Supplementary file 1 — Supplementary Material 1 [file 12989_2024_568_MOESM1_ESM.docx]
